# Co-Application of Silicon with Selenium, Sulphur, Zinc, and Iron in Plants: Mechanisms of Stress Tolerance, Nutrient Homeostasis and Secondary Metabolism

**DOI:** 10.3390/plants15162463

**Published:** 2026-08-14

**Authors:** Marija Polić Pasković, Mohammed Bouhadi, Soukaina Lahmaoui, Igor Pasković

**Affiliations:** 1 Institute of Agriculture and Tourism, Karla Huguesa 8, 52440 Poreč, Croatia; paskovic@iptpo.hr; 2 Laboratory of Biotechnology, Conservation and Valorization of Bioresources, Department of Biology, Faculty of Sciences Dhar Mehraz, Sidi Mohamed Ben Abdellah University, Fez 30000, Morocco; bouhadi.mohammed@usmba.ac.ma

**Keywords:** beneficial elements, biofortification, heavy metal toxicity, antioxidant defence, nanoparticle fertilisers, drought and salinity tolerance

## Abstract

While individual Si-nutrient interactions have been reviewed separately, a comparative analysis of multiple Si-element interactions remains lacking. This review compares current knowledge on co-application of Si with selenium (Se), sulphur (S), zinc (Zn) and iron (Fe), focusing on stress tolerance, nutrient homeostasis, physiological responses, secondary metabolism and agronomic relevance. The evidence indicates that combining Si with these elements helps maintain reactive oxygen species (ROS) homeostasis, strengthen antioxidant defenses, stabilize photosynthetic function and improve nutrient uptake, translocation and use efficiency. Responses depend on plant species, nutrient form, application strategy and environmental conditions; at the metabolic level, Si-based combinations affect the synthesis of phenolic compounds, amino acids and sulphur-containing metabolites. Si-Se and Si-Fe proved most effective under heavy-metal stress, through regulation of metal transport, detoxification and sequestration, and Si-S and Si-Zn under drought, salinity and nutrient-deficient conditions, by enhancing osmotic adjustment, nutrient-use efficiency, ionic homeostasis and photosynthetic performance. Agronomically, these interactions can increase crop productivity, nutritional value and biofortification potential, and mitigate toxic-element accumulation in edible parts. Knowledge gaps remain regarding molecular regulation, variability among species and environments, and the long-term effectiveness of nanoparticle formulations. Since most evidence comes from hydroponic, pot and greenhouse studies, standardized field experiments are needed to assess agronomic relevance.

## 1. Introduction

Plants face a wide range of environmental constraints that strongly influence their growth, development, metabolism, and productivity. In general, abiotic stresses such as drought, salinity, extreme temperatures, heavy metal stress, and nutrient imbalances are present, and biotic stresses, including pathogen infection, insect attack, and other biotic stresses, are also present [1]. Abiotic stresses are one of the major constraints to agricultural production as they affect photosynthesis, water relations, nutrient uptake, redox homeostasis, and biomass accumulation and result in significant yield losses [2]. At the same time, biotic stresses also decrease plant performance and productivity by imposing high metabolic costs associated with defense responses [3,4,5,6]. Combined, these stresses affect both primary and secondary metabolism, resulting in decreased plant growth, survival, and crop productivity [7].

The constraints may become more severe with increasing climate variability, land degradation, and water shortage; therefore, there is a strong need to develop sustainable approaches to enhance the resilience of crops and ensure food security [8]. Modern agriculture is therefore moving toward sustainable strategies that can increase plant resistance while reducing the use of synthetic chemicals [9,10]. In this context, beneficial mineral elements and integrated nutrient management have become an increasing focus of interest as a potential tool to enhance plant performance under optimal and stressful conditions.

Silicon (Si) has attracted increasing attention as a beneficial element for improving plant resistance to environmental stresses. Si is not an essential element for most plant species, but it is the second most abundant element in the earth’s crust, and is primarily taken up by the plants as monosilicic acid [11,12]. After uptake, Si can be deposited in epidermal tissues and cell walls, contributing to the strengthening of physical barriers and improving plant resistance against several environmental constraints. In addition to this structural role, Si also regulates various physiological and biochemical processes, such as photosynthetic efficiency, water-use balance, nutrient homeostasis, activation of the antioxidant defense system, and metabolic reprogramming in response to stress [13,14]. There are the following different methods of application that can be used to provide silicon to plants: soil amendment, hydroponic silicon supplementation, foliar silicon application, and seed priming. It comes in various types, including sodium silicate, potassium silicate, calcium silicate, and silicon nanoparticles (SiNPs), which vary in solubility, bioavailability, and agronomic suitability [15,16]. Silicon’s ability to be applied and formulated in different ways makes it a very versatile and environmentally friendly tool for improving crop productivity and stress tolerance. Therefore, a number of studies have reported that Si alleviates the negative responses of drought, salinity, heavy metal toxicity, nutrient deficiency, and pathogen attack, mainly through improving antioxidant enzyme activities, reducing the accumulation of reactive oxygen species, maintaining membrane stability, and improving nutrient-use efficiency [13,14,17].

The elements such as nitrogen (N), phosphorous (P), potassium (K), calcium (Ca), magnesium (Mg), sulphur (S), iron (Fe), and zinc (Zn) are essential components involved in plant growth, photosynthesis, enzymatic activity, osmotic regulation, redox balance, and secondary metabolism, whereas selenium (Se) is considered a beneficial element that can improve plant growth and stress tolerance under specific conditions [18,19]. Still, nutrient deficiency, nutrient imbalance, low soil fertility, and low nutrient availability are major constraints to global crop production [20,21]. Therefore, the use of Si in combination with other elements is a particularly promising approach in crop production, as Si has been demonstrated to control nutrient availability, uptake, translocation, and homeostasis in various plant species and environmental conditions [22,23]. While Si also interacts with other essential macro- and micronutrients, including N, P, K, Ca, and Mg, the present review deliberately narrows its focus to Se, S, Zn, and Fe, without implying that these other interactions are of lesser physiological relevance. This selection is supported by three complementary criteria. First, Se, S, Zn, and Fe share a common functional link with Si centered on redox homeostasis, antioxidant defense, and the biosynthesis of secondary metabolites, including phenolics, which constitute the central physiological and metabolic axis of this review [23,24]. Sulphur is further connected to this axis through its metabolic coupling with selenium, as Se assimilation in plants directly affects sulphur metabolic pathways and the biosynthesis of S-containing compounds [25]. In contrast, the interaction of Si with K is primarily associated with osmotic regulation and water relations under stress conditions [24], while Si–Ca interactions are mainly linked to structural and membrane-level processes; these mechanisms, although physiologically important, fall outside the redox- and secondary metabolism-centered scope adopted here and are therefore only briefly noted for comparative context rather than reviewed in depth. Second, Se, Zn, and Fe are of direct relevance to human nutrition, as their deficiencies represent major global micronutrient malnutrition concerns, while Se and S together influence the nutritional quality of S-containing secondary metabolites relevant to both plant defense and human diet [26,27]; consequently, Si-mediated improvement of the uptake, translocation, and use efficiency of these elements holds particular promise for agronomic biofortification strategies [28,29,30]. Third, although interest in these specific Si-nutrient combinations has increased considerably in recent years, the corresponding literature remains scattered across individual studies rather than synthesized within a comparative framework. Together, this combination of shared physiological mechanisms, nutritional relevance, and literature gap supports the rationale for focusing this review on Si interactions with Se, S, Zn, and Fe and their downstream effects on primary and secondary metabolism, as discussed below.

Based on this nutritional foundation, studies on the interactions between co-application of Si and selected mineral elements have revealed a variety of interactions with possible functional implications. The Si/Si + S combination protects against oxidative stress and redox regulation of S-containing compounds [25]; Si/Zn enhances photosynthesis, gas exchange, and growth under water deficit [31] in terms of antioxidant protection and redox status. Regarding mineral homeostasis and structural integrity, Si interacts with Fe to promote chlorophyll biosynthesis and alleviate metal toxicity [32]. The combination of Se and Si has attracted growing interest or its synergistic effects in antioxidant protection and crop biofortification [33].

The effect of Si and mineral elements is not limited to nutrient uptake and plant growth; they also influence plant metabolism. The secondary metabolism is closely associated with plant adaptation to biotic and abiotic stresses, which can be modulated by silicon [24]. The secondary metabolites, such as phenolic compounds, flavonoids, terpenoids, and glucosinolates, are involved in ROS detoxification, pathogen resistance, osmoprotection, and stress signaling [31,32]. Silicon–mineral co-application can help control the redox status, enzyme activity, and important metabolic pathways, and thus support the biosynthesis or accumulation of these bioactive compounds, such as phenolics and terpenoids affected by Se [34] and S-containing metabolites influenced by Se-S interaction [33]. This is especially pertinent to enhancing stress tolerance capacity and the crop quality, nutritional value, and biofortification potential of combined Si and other elements [25,35].

While there are numerous studies that examined the individual effects of Si or other nutrients on plant growth and stress tolerance, their combined effect with Si has not been systematically studied yet [23,36,37]. The mechanisms of action, nutrient interactions, and secondary metabolic changes resulting from Si+ combinations should also be better understood, as the response of the plant to Si-mediated changes in nutrient availability, uptake, translocation, and homeostasis can differ between plant species, growth conditions, and soil properties [22,23,24]. These interactions are fundamental for the implementation of efficient fertilization and biostimulant strategies suitable for sustainable agriculture [38,39].

This review was prepared as a narrative synthesis of current knowledge, focusing on recent and relevant studies dealing with the co-application of Si with Se, S, Zn, and Fe in plants. The selected literature includes experimental studies and key review papers related to Si availability, uptake, transport, stress tolerance, nutrient homeostasis, biofortification, and modulation of primary and secondary metabolism. Although emphasis was placed on recent research, earlier foundational studies were included where necessary to provide additional background. Thus, this review provides an overview of the co-application of Si with selected essential and beneficial elements in plants and emphasizes physiological responses, stress tolerance mechanisms, nutrient balance, and modulation of secondary metabolism. Special focus is placed on potential complementary, additive, or synergistic interactions of Si with Se, S, Zn, and Fe and their agronomic relevance for improving crop productivity, nutritional quality, and environmental sustainability under varying environmental conditions.

This review adopts a narrative rather than a systematic approach. The literature was primarily retrieved through Google Scholar, ScienceDirect, Scopus, and Web of Science, using combinations of keywords related to silicon, selenium, sulphur, zinc, iron, stress tolerance, nutrient homeostasis, biofortification, and secondary metabolism. Peer-reviewed research articles and review papers were selected based on their relevance to the objectives of this review, with particular emphasis on studies addressing Si co-application with Se, S, Zn, and Fe, as well as their effects on plant physiology, stress tolerance, nutrient interactions, and metabolic regulation.

## 2. Silicon Availability, Uptake, Transport, and Accumulation in Plants

### 2.1. Silicon Forms and Availability in Soil

**Figure 1:**
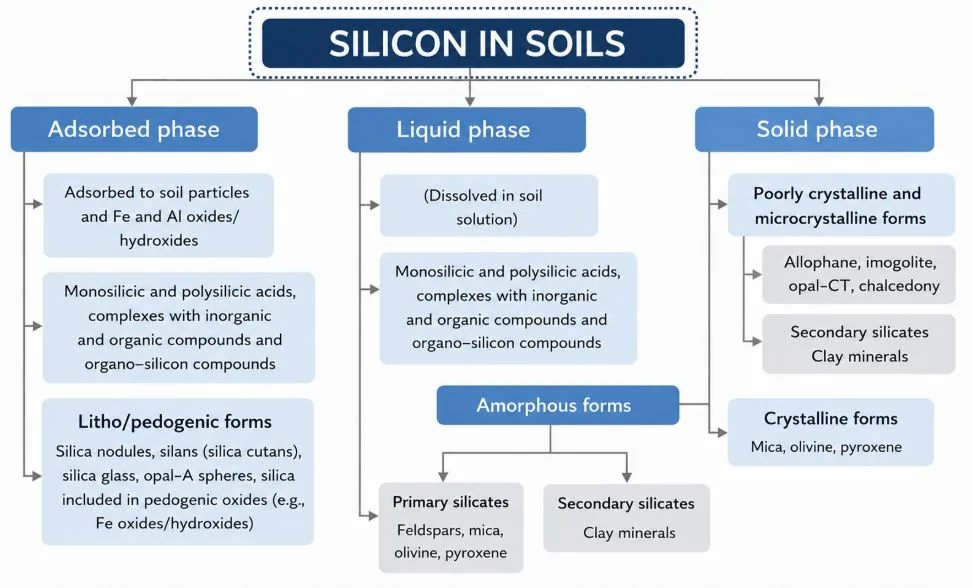
Main fractions and forms of silicon in soils.

Silicon (Si) is the second most abundant element in the Earth’s crust after oxygen, and it constitutes about 28% of the Earth’s crust [26,27]. In soil, the most common silicon compounds are silicates and silicon dioxide, with soil Si content ranging from 50 to 400 g kg^−1^, varying with soil type, texture, and mineral composition [40,41]. Although Si is abundant in soil, the vast majority is present in non-dissolved solid crystalline or amorphous forms, making it unavailable for direct uptake by plants [42]. The distribution of soil Si is mostly in the solid, liquid, and adsorbed phases, and mineral weathering, dissolution, adsorption–desorption reactions, and degradation of biogenic materials such as plant residues and microbial remains are the controlling processes for its release into the soil solution [42,43] (Figure 1). In soil solution, Si is mainly present as monomeric silicic acid [Si(OH)_4_], which is the only form that can be directly taken up by the roots, while polymerized forms like polysilicic acids are generally not directly assimilable [44,45]. In contrast, Si compounds are more poorly soluble, and the concentration of plant-available Si in soil solution is relatively low (typically 0.1–0.6 mM) [42,44,46]. This indicates that the availability of Si to plants is not only strongly dependent on its total content in soil but also on soil pH, mineral composition, organic matter content, biological activity, and dynamic balance among the solid and soluble pools of Si in soil [43].

### 2.2. Silicon Uptake and Transport Mechanisms

Silicon is considered a beneficial element and is not included in the current classification of essential macro- or micronutrients in plant nutrition [47]. However, Si deficiency may negatively affect plant growth, development, and reproduction, and some authors therefore classify it as a “quasi-essential” element [48]. In higher plants, Si uptake, transportation, and accumulation depend on the concentration of water-soluble monomeric silicic acid [Si(OH)_4_] in soil solution, the activity of certain Si carriers, and Si transport via transpiration in the xylem pathway [49,50,51,52]. While all plants have very little silicon in their dry biomass, there are considerable differences between species concerning the Si uptake, transportation, and deposition in different plant organs [44,53,54,55].

Plants take up Si mainly from the soil solution in the form of monomeric silicic acid [Si(OH)_4_]. The first Si influx transporter, Low Silicon 1 (*Lsi1*), was identified in rice (*Oryza sativa* L.) through forward-genetic mutagenesis and shown to facilitate the entry of silicic acid from the external medium into root cells [56]. This discovery demonstrated that Si uptake in rice is mediated by a specific transport system and can occur more rapidly than water uptake [57]. *Lsi1* is part of the aquaporin family called Nodulin 26-like Intrinsic Protein (NIP) and is expressed consistently in both primary and lateral roots, especially in the exodermis and endodermis. *Lsi1* is located on the distal side of the cell’s plasma membrane. Interestingly, *Lsi1* is not found in root hairs [49,50]. The Casparian bands in the exodermis and endodermis prevent apoplastic movement of solutes; hence, the movement of Si through root tissues is mainly reliant on the symplastic and transcellular pathways [51]. In these pathways, aquaporins are important for water and nutrient transport, especially when plants are under drought and salt stress conditions [52,58]. A second transporter, *Lsi2*, is a Si efflux transporter found on the same exodermal and endodermal root cells, actively pumping Si out from the cells to the stele [59]. In contrast to *Lsi1*, *Lsi2* is an active transport protein that is linked to proton-ATPase activity, resulting in an H^+^ gradient [59]. The polar, non-overlapping localizations of Lsi1 and Lsi2 thus create a transcellular relay that drives directional Si flux from the rhizosphere toward the vascular cylinder.

Once in the stele, Si is introduced into the xylem using the silicon efflux transporter, *Lsi3*, which occurs within the pericycle cells of rice roots and lacks polarity [60]. Subsequently, Si is transported to the shoot organs using the transpiration stream. In the case of rice, the unloading of Si from the xylem into the shoot tissues involves another transporter, namely *Lsi6*, the homolog of *Lsi1* and a member of the same NIP III subfamily of aquaporins, located in the xylem parenchyma cells, leaf sheath, and leaf blade [61]. During the intervascular transport process at the node level, three different transporters—*Lsi2*, *Lsi3*, and *Lsi6*—work together to facilitate Si transfer, thus ensuring the selective transport to plant tissues, which is required for efficient Si accumulation in the reproductive tissues of rice plants [62].

Since then, Si transporter homologs have been found to exist not only in the studied cereal crops but also in other monocotyledonous plants including barley, wheat, maize, and sorghum, as well as some dicotyledonous plants such as pumpkin and cucumber [63,64,65,66,67]. Notably, the protein Lsi1 was shown to be expressed in cucumber root hairs but not in rice [65]. Nonetheless, certain important points about Si transport processes still remain unknown, particularly regarding its delivery into silica cells [50].

### 2.3. Silicon Accumulation Among Plant Species

Silicon accumulation varies widely among plant species, with the range of accumulation varying between 0.1% and 10% of plant dry weight, especially within the transpiring organs [50]. Plants can be grouped into the following three categories based on their silicon concentration: high accumulators, medium accumulators, and low accumulators [41]. Although all plant species contain some level of Si in their dry matter, they differ significantly in their capacity to accumulate Si through root uptake. Consequently, plants are categorized as hyperaccumulators (>1% Si in dry matter), intermediate accumulators (0.5–1% Si in dry matter), and Si non-accumulators (<0.5% Si in dry matter) [44,53,54,55]. According to other authors, the threshold values for this classification are considerably higher, distinguishing hyperaccumulators with >4% Si in dry matter (plants of the orders *Equisetales*, *Poales*, and *Cyperales*), intermediate accumulators with 2–4% Si in dry matter (*Cucurbitales* and *Urticales*), non-accumulators with 0.5–2% Si in dry matter, and plants that do not accumulate Si with <0.5% Si in dry matter (*Solanum lycopersicon* L.). In addition, plants can be distinguished according to the rate of Si uptake relative to water flux; species absorbing Si at rates exceeding water uptake are classified as active accumulators (rice, wheat, and barley), those absorbing it at similar rates as passive accumulators (oat), and those absorbing it at lower rates as Si excluders [57].

Typically, Si uptake has been associated with monocots, especially grasses, including rice, wheat, barley, and maize, which exhibit high to intermediate Si uptake, while Si uptake has been shown to be low for the majority of dicots [68]. Nevertheless, it should be noted that Si uptake is not only determined by taxonomy since it requires active genes of the Lsi1 orthologue for effective uptake; thus, some dicotyledonous plants can accumulate more Si than some monocotyledonous. Following its transport and uptake via xylem, Si deposition occurs in plants mainly as amorphous silica or phytoliths as a consequence of the biosilicification process through the condensation of Si(OH)_4_ molecules to form SiO_2_·nH_2_O upon the saturation point of silicic acids above their solubility capacity [24,50,69]. In grass species, about 90% of Si taken up by the plants is retained in the epidermis of the leaf and the husk of grains, which involves the action of transpiration flow in creating silica layers under the cuticles, making them stiff and resistant [12,14,70]. Leaf age affects Si deposition such that aged leaves contain more Si than young ones, which are localized only in specific bulliform cells, while senescent leaves have Si in almost all kinds of cells [50]. Concentrations of Si in the aboveground parts of different agricultural crops were compared by [63,71,72,73,74], including rice (*Oryza sativa* L.), wheat (*Triticum aestivum* L.), pumpkin (*Cucurbita maxima* Duch.), zucchini (*Cucurbita pepo* L.), chickpea (*Cicer arietinum* L.), cucumber (*Cucumis sativus* L.), and maize (*Zea mays* L.), with reported values ranging from 3000 to 39,100 mg kg^−1^ dry matter. Hodson et al. [75] reported an average Si concentration of 320 mg kg^−1^ in the green, non-woody tissues of olive.

Physiological availability of Si in plant tissues is determined by a combination of mechanisms as follows: the control of Si availability and dissolution in soil [42,43], its uptake and transcellular transport in the root through the Si transporter system Lsi1–Lsi2–Lsi3–Lsi6 [49,50,59,60,61] and species-dependent accumulation/deposition patterns [44,53,54,55]. Therefore, these properties are related to transport and accumulation and are critical to the understanding of the interaction of Si with selenium, sulphur, zinc and iron, which affects their uptake, translocation and homeostasis, as discussed in the following sections.

## 3. Functional Roles and Application Strategies of Silicon in Plants

### 3.1. Silicon in Plant Stress Tolerance

Silicon confers resistance to a wide range of biotic and abiotic stresses through both structural and physiological effects [76,77,78]. Under biotic stress it acts in the following two ways: Si deposition beneath the leaf cuticle creates a mechanical barrier that hinders fungal penetration and reduces the digestibility of leaf tissue to insects [36,79], and Si triggers biochemical defense responses, resulting in accumulation of lignin, phenolic compounds, phytoalexins and defense-related enzymes such as peroxidases and phenylalanine ammonia-lyase (PAL) [31,74]. Si is also involved in jasmonic acid, salicylic acid and ethylene signaling pathways that strengthen defense against pathogens and pests [74,75].

Under abiotic stress, Si improves root hydraulic conductivity, leaf relative water content and water-use efficiency under drought and salinity [80,81,82,83,84,85,86]. It contributes to osmotic adjustment by increasing compatible solutes (proline and glycine betaine) and reduces ROS accumulation, lipid peroxidation and membrane damage by stimulating antioxidant defense systems [87,88,89,90]. Si further maintains photosynthetic activity and biomass accumulation and enhances tolerance to heavy metals and UV-B through nutrient homeostasis and cellular protection [13,91,92,93,94].

Growing evidence indicates that these effects can be enhanced when Si is applied together with other mineral nutrients. Combined Si–K supplementation improved cadmium and lead tolerance in quinoa beyond either treatment alone [95] and alleviated salt stress in passion fruit seedlings through improved nutrient uptake and reduced Na^+^ accumulation [96]. Comparable benefits have been reported for Si–Zn under drought in *Eruca sativa* L. [97] and for Si–Ca–K in cotton under water deficit [98]. Si therefore acts not only as a stress-mitigating element but as a component of integrated nutrient-management strategies.

### 3.2. Silicon Application Methods

Silicon is supplied to plants through soil application, foliar spray, fertigation, hydroponic supplementation or seed priming, most often as sodium silicate (Na_2_SiO_3_) or potassium silicate (K_2_SiO_3_) [99,100]. Foliar application is particularly effective against fungal diseases: it controls powdery mildew in cucumber, melon, zucchini, pumpkin, strawberry and grapevine, as well as downy mildew and gray mold in strawberry and blue mold in several fruit crops [101], effects attributed to enhanced physical barriers and increased synthesis of antimicrobial phenolic compounds [102]. In olive, foliar potassium silicate reduces olive leaf spot (Venturia oleaginea), promotes vegetative growth [103,104] and improves nitrogen and potassium uptake in young trees [104,105]; Si particle films are widely used to deter pests such as the olive fruit fly [106], and kaolin application additionally enhances heat tolerance, photosynthetic activity and water status under drought [107]. Si application also alters the phenolic composition and quality of olive [101,108,109].

Si is also effective in combination with biological strategies: foliar Si applied with salt-tolerant plant growth-promoting rhizobacteria (PGPR) improved antioxidant enzyme activities and reduced oxidative damage in Pak choi under salinity [110,111].

### 3.3. Silicon in Biofortification Strategies

Biofortification is considered an agronomic approach for enhancing the content and bioavailability of target nutrients in edible plant tissues while simultaneously improving plant response to stress conditions [112]. Selenium (Se) biofortification has gained a particular interest since the biofortification of plants with inorganic Se is converted into organic Se that is more bioavailable to humans than inorganic Se supplementation [28,29,30,113,114,115,116,117,118,119]. Beyond nutrient supply, the success of biofortification relies on the physiological mechanisms that control nutrient uptake, transport, assimilation and storage in plant tissues [35,120]. Therefore, interactions between target nutrients and beneficial nutrients that are essential for plant growth and nutrient homeostasis are of great importance to the biofortification efficiency [121]. Si, though not an essential element to the growth of most higher plants, has beneficial effects on mineral nutrition, antioxidant metabolism, redox homeostasis, photosynthetic performance and abiotic stress tolerance [82,122]. In these ways, Si generates physiologically beneficial conditions for the improved absorption, transport, accumulation, and use of nutrients [17].

Hence, the contribution of Si to biofortification is not restricted to its direct physiological effects. Si enhances the efficiency of nutrient uptake, stress resistance and cell protection which allows the successful deposition of nutrient elements in plants. This understanding is crucial to designing biofortification strategies that are more efficient and sustainable in the context of interactions between Si and key nutrients like selenium, sulphur, zinc and iron. These interactions form the main focus of the subsequent parts of this review.

## 4. Silicon Interactions with Selenium, Sulphur, Zinc, and Iron in Plants

Although Si is not classified as an essential nutrient for higher plants, an increasing body of evidence indicates that it plays a crucial role in modulating plant responses to environmental stress and nutrient homeostasis. In recent years, particular attention has been given to the combined application of Si with other beneficial or essential elements, as such interactions may induce synergistic physiological and metabolic responses in plants. Among these, the interactions between Si and elements such as selenium (Se), sulphur (S), zinc (Zn), and iron (Fe) are emerging as particularly relevant due to their shared involvement in redox regulation, antioxidant defense systems, and nutrient biofortification strategies. Understanding these interactions is increasingly important in the context of sustainable agriculture and climate-driven stress conditions, where combined nutrient management may provide an effective strategy to enhance plant resilience and crop nutritional quality. Despite the growing number of studies on Si nutrition, the mechanisms and agronomic implications of its combined application with selected essential and beneficial elements remain poorly synthesized.

### 4.1. Silicon and Selenium

Plants are the main source of Se in human diets and animal feed. Selenium deficiency in the diet represents a significant global problem that can lead to serious health consequences. It is estimated that insufficient Se intake affects up to one billion people worldwide [123]. In China and Russia, endemic diseases such as Keshan disease, a cardiomyopathy, and Kashin–Beck osteoarthritis have been identified and are directly associated with Se deficiency in the human diet. In Finland, biofortification of crops with Se has been used since the 1960s in animal nutrition and human diets as a preventive measure due to the low Se content in soils [124]. After twenty years of research on Se application in Finnish crops, Aspila [125] concluded that sodium selenate applied foliarily is the most suitable method for Se biofortification.

In the human body, Se deficiency may lead to disorders of the endocrine, musculoskeletal, cardiovascular, immune, reproductive, and nervous systems. Recent studies indicate that adequate Se levels in the body may reduce the risk of certain cancers, male infertility, viral infections, mood disorders, and cardiovascular diseases [126]. Recommended Se intake for adults differs between men and women and also varies worldwide. For men, recommendations range from 40 μg day^−1^ according to the World Health Organization (WHO) to 85 μg day^−1^ in Australia, while for women the recommended intake ranges from 30 μg day^−1^ according to WHO to 70 μg day^−1^ in Australia. WHO has also estimated the basal requirement for Se intake—the amount needed to prevent pathological and clinically significant signs of nutritional deficiency—at 21 μg day^−1^ for men and 16 μg day^−1^ for women, while a daily intake of 400 μg is considered toxic for both sexes [127]. Excessive intake causes nausea, vomiting and diarrhea, and chronically leads to selenosis, damaging the cardiovascular, gastrointestinal, neurological and hematological systems [128]. Previous studies conducted in Croatia have identified subadequate Se levels in fruits and cereals produced in eastern Croatia [129]. They also showed that the average daily dietary intake of Se in eastern Croatia is inadequate, amounting to 27.3 μg day^−1^ [130]. In addition to the positive effects of individual foliar treatments with Se and Si, numerous studies have shown that the combined biofortification with these elements induces phytohormonal and antioxidant stress-response mechanisms in various plant species [131]. The authors emphasized that the interaction between Se and Si activates similar signaling pathways in many plants, although their efficiency varies among species.

It has also been shown that the foliar application of Se or Si nanoparticles, as well as the foliar application of potassium silicate, increases phenolic production in strawberry (*Fragaria ananassa* L.) under stress conditions [113] or the concentration of phenolic compounds in olive leaves grown under field conditions without irrigation [108]. Similarly, the combined foliar application of Se and Si has been reported to modulate both the phenolic profile and amino acid composition in olive leaves grown under rainfed field conditions, suggesting a broader impact on plant primary and secondary metabolism [132]. Phenolic compounds are well known for their antioxidant properties and their significant role in human nutrition. Their presence in fruits, vegetables, and other food products contributes to the protection of cells from oxidative stress, thereby reducing the risk of chronic diseases such as cardiovascular diseases, cancer, and neurodegenerative disorders [133].

In plants, phenolic compounds play a key role in metabolic plasticity, i.e., the ability of a species to adjust its primary and secondary metabolism in order to acclimate (and potentially adapt) to highly variable environmental conditions. Global climate change is imposing a general increase in temperatures and a decrease in precipitation in the Mediterranean regions, accompanied by a higher frequency of extreme climatic events such as heat waves and late spring frosts. High solar radiation, which often leads to stress, represents one of the first challenges plants face in most Mediterranean environments. The ability to absorb shorter wavelengths of solar radiation is characteristic of many phenolic compounds, including the absorption of UV-A and UV-B radiation [134]. Phenolics also trigger a network of events, including stress-induced morphogenesis, which protects plants from further and unexpected damage of different origins. Agati et al. [135] suggested that the signals activating flavonoid biosynthesis include drastic changes in ROS homeostasis, which explains the temporal correlation between flavonoid biosynthesis and oxidative stress events. Di Ferdinando et al. [134] concluded that phenolic compounds have a strong capacity to prevent ROS formation, neutralize ROS once they are formed, and precisely regulate key steps of cellular growth and differentiation [133].

**Figure 2:**
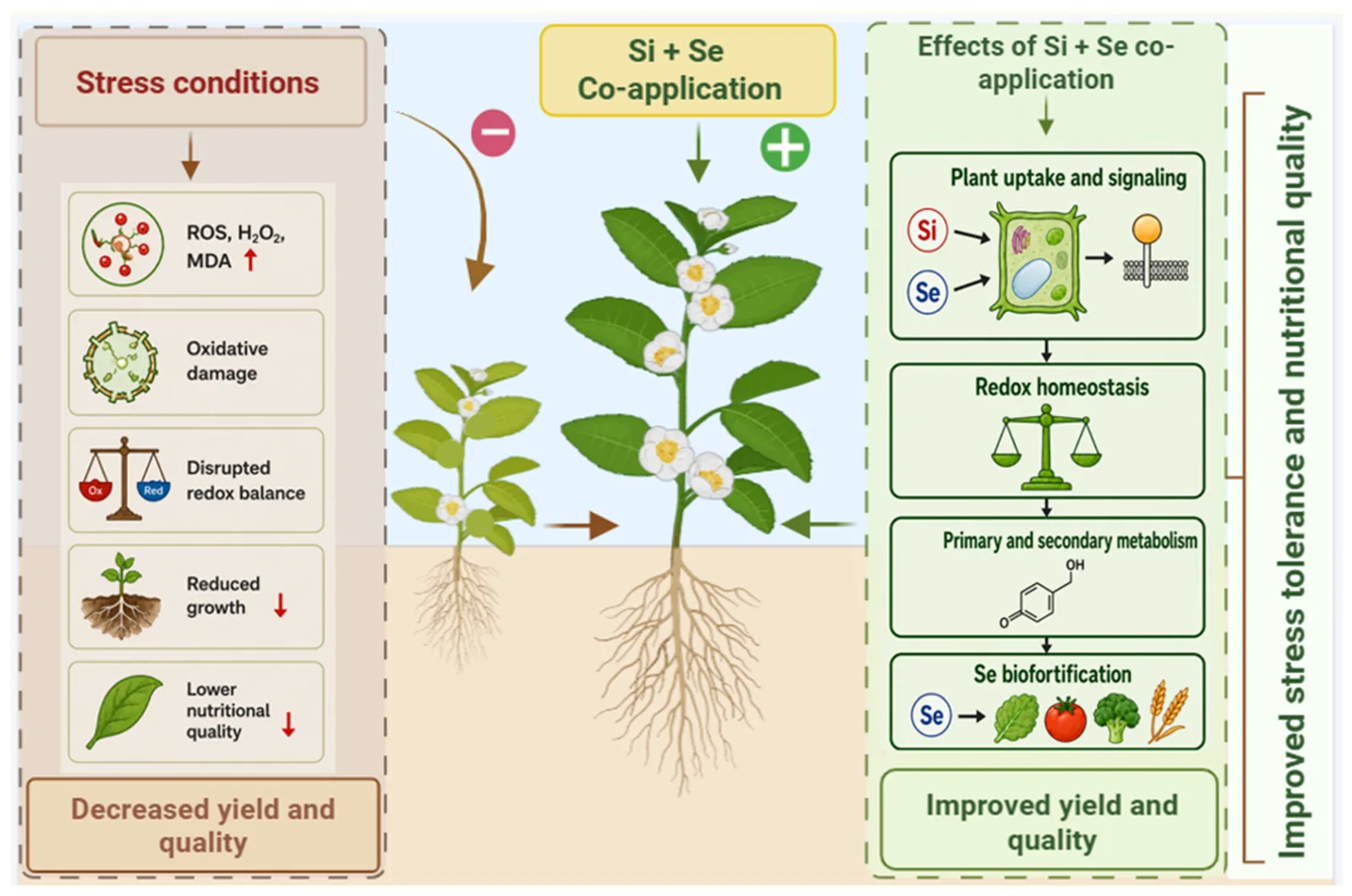
Effects of silicon (Si) and selenium (Se) co-application as a strategy to alleviate stress in plants: mechanisms of action from cellular signaling and redox homeostasis to enhanced primary and secondary metabolism, Se biofortification, and improved yield and nutritional quality (created with BioRender.com).

Although numerous studies report beneficial effects of combined Se and Si application, the magnitude and nature of these responses are highly dependent on crop species, genotype, stress conditions, selenium and silicon forms, and application methods. Strong synergistic effects have frequently been observed under salinity, drought, heat, and heavy-metal stress, where Se and Si jointly improve antioxidant defenses, osmotic adjustment, photosynthetic performance, and ion homeostasis [28,113,114,118]. However, the interaction is not always synergistic. For example, in tomato, combined Se–Si application mainly produced additive effects, with Si contributing predominantly to vegetative growth and yield, whereas Se was more effective in improving fruit nutritional quality [136]. Similarly, cultivar-dependent differences were reported in rice under Cd stress, where the efficiency of combined Se–Si treatments varied significantly among genotypes [137]. Variability has also been associated with the formulation used. While Se and Si nanoparticles often improve stress tolerance and biofortification efficiency, hetero-aggregation between nano-Se and nano-Si may reduce nanoparticle uptake and lower treatment efficiency, as reported in common bean and wheat [138]. In addition, application methods have a significant impact on treatment efficiency, and foliar application can lead to accumulation of Se, antioxidant activity, and photosynthetic efficiency, while soil and hydroponic application is more likely to influence nutrient availability, ion transport, metal immobilization, and root sequestration mechanisms [28,117,118]. The results demonstrated that Se–Si interactions are not always synergistic and should be optimized based on the crop species, genotype and environmental conditions. An overview of studies investigating the combined application of Se and Si and their effects on other agricultural crops is presented in Table 1 and Figure 2. Most studies on combined Si and Se application have been conducted in cereals, particularly rice and wheat, while research on woody and herbaceous crops remains limited.

### 4.2. Silicon and Sulphur

**Figure 3:**
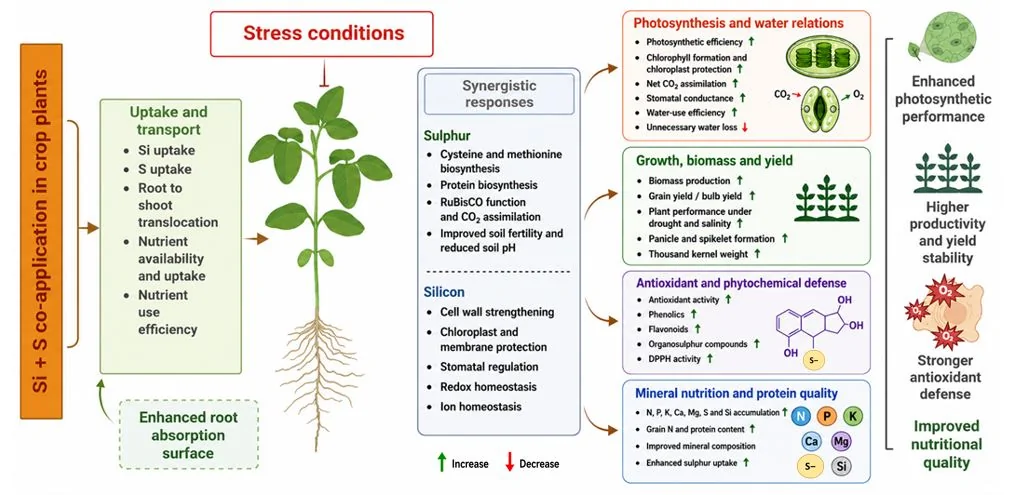
Schematic representation of the complementary mechanisms of silicon (Si) and sulphur (S) co-application in crop plants under stress (created with BioRender.com).

The synergistic application of sulphur (S) and silicon (Si) represents a sophisticated approach to enhancing crop performance, particularly when plants are subjected to environmental stress. Research underscores a multi-faceted benefit to this elemental combination, ranging from physiological resilience to improved nutritional profiles (Table 2). For instance, in oilseed crops such as canola (*Brassica napus* L.), the soil application of nano-silicon paired with sulphur has been shown to significantly bolster drought resilience. According to Alizadeh et al. [151], this specific synergy enhances photosynthetic efficiency and nutrient uptake, which consequently drives increased biomass production under water-limited conditions. At the physiological level, sulphur contributes to the synthesis of sulphur containing amino acids, such as cysteine and methionine, which are important for RuBisCO function and CO_2_ assimilation [152]. In parallel, silicon strengthens cell walls, improves leaf architecture, protects chloroplasts and cellular membranes, and supports photosynthetic efficiency, stomatal regulation, and redox homeostasis [153]. The complementary roles of sulphur and silicon in improving plant performance, stress tolerance and nutritional quality are summarized in Figure 3.

In addition to the drought stress, the beneficial effect of S and Si has also been observed under saline soil conditions. Elemental sulphur and potassium silicate supplementation was found to enhance the grain yield, leaf mineral composition, grain nitrogen content, grain protein content, as well as soil S and Si availability in maize, *Zea mays* L. [154]. These effects suggest that sulphur and silicon act through complementary mechanisms. Sulphur contributes to soil fertility and plant nutrition by improving nutrient availability and uptake, particularly in saline or alkaline soils. This effect is mainly related to its ability to reduce soil pH and improve soil chemical properties, as reported by Kineber et al. [155] in alkaline soils. Similarly, *Thiobacillus* bacteria can oxidize sulphur to sulphuric acid, which reduces soil pH in calcareous soils and consequently enhances the availability of nutrients such as phosphorus, potassium, and calcium [156].

Sulphur is also involved in nitrogen metabolism and in protein biosynthesis through the formation of sulphur-containing amino acids like cysteine and methionine.

In contrast, silicon acts mainly as a stress-mitigating element by protecting chloroplasts and cellular membranes, maintaining photosynthetic activity, and supporting ion homeostasis under salt stress [157]. This protective effect may be related to the activation of membrane transport systems such as H^+^-ATPase and H^+^-PPase—essential for maintaining the ionic balance in cells under salinity stress [158]. Similarly, findings in cereal crops further validate the efficacy of this integrated nutritional strategy. In the case of winter oats (*Avena sativa* L.), foliar applications of Si and S have proven to be highly effective. Specifically, Kutasy et al. [159] demonstrated that this combination boosts water-use efficiency by reducing unnecessary water loss and maintaining high photosynthetic rates during drought. Moreover, the benefits extend beyond mere yield stability. In addition to enhancing survival mechanisms, this elemental pairing serves a vital biofortification role. For example, Kutasy et al. [160] observed significant improvements in the mineral composition and protein content of oats, a result largely driven by enhanced sulphur uptake. More recently, Forgács et al. [161] confirmed the agronomic relevance of combined Si and S foliar fertilization in winter oat, showing that the Si + S treatment gave the highest grain yield, reaching 5796.47 kg ha ^−1^. Interestingly, not all individual yield components were optimized with the combined treatment; sulphur alone mainly increased the number of panicles per square meter and the number of spikelets per panicle, while silicon alone increased thousand kernel weights. This suggests that the positive effect of Si + S is independent of any single yield component and is rather due to the synergistic action of both elements, where sulphur plays a role in mineral nutrition, reproductive growth, and silicon plays a role in physiological stability and stress tolerance.

The complementary action of Si and S has also been demonstrated in garlic (*Allium sativum* L.). The use of both silicon and sulphur led to an increase in the amount of P, K, and Mg in the bulbs and an increase in antioxidant and phytochemical defense [162]. The highest DPPH antioxidant activity was obtained under combined treatment, which was related to the stimulation of Si-related phenolic accumulation and organosulphur compounds dependent on S.

In addition to the agronomic advantages, Si and S act synergistically to regulate sulphur metabolism and antioxidant defense. Sulphur is an essential element in the structure of cysteine, methionine, glutathione (GSH), and a number of sulphur-containing metabolites that are important in redox regulation and adaptation to stress. Sulphur availability stimulates GSH production and the ascorbate–glutathione cycle, which in turn increases the detoxification of reactive oxygen species (ROS) and cellular protection. These functions are complemented by silicon, which can help against oxidative damage, maintain membrane stability, protect chloroplast structure and trigger the activity of antioxidant enzymes like superoxide dismutase (SOD), catalase (CAT), and ascorbate peroxidase (APX) [151,162]. In canola, combined Si-S application was found to significantly increase the antioxidant capacity and photosynthetic performance and to decrease lipid peroxidation, further supporting this [162]. Similar synergistic benefits of combined silicon–sulphur nutrition on growth, yield, and biochemical quality have also been reported in rice [163,164] and vegetable soybean [165]. Taken together, these results suggest that sulphur is mainly involved in the synthesis of antioxidant metabolites, while silicon stimulates their protective action by controlling the integrity of cells and their physiological equilibrium, which leads to increased tolerance to stress and plant growth.

However, it is the strategic combination of these two elements—rather than their individual application—that appears to be the key driver of these results. Ultimately, these studies suggest that integrating Si and S is an essential tool for modern agriculture to ensure both food security and high crop quality.

**Table 2:** Overview of recent studies on the application of sulphur (S) and silicon (Si) and their effects on agricultural crops.

Plant Species	Stress Condition	Sulphur Source (Dose + Application)	Silicon Source (Dose + Application)	Main Physiological Responses	Affected Metabolites/Molecular Responses	Proposed Mechanism	Ref.
Canola *(Brassica napus* L.)	Water deficit stress (0.8, 0.6, and 0.4 field capacity)	Potassium sulfate; 0, 75, and 150 mg S kg^−1^ soil, soil application	Silicon nanoparticles (Si-NPs); 0, 100, 200, and 300 mg kg^−1^ soil, soil application	Increased shoot and root biomass, chlorophyll and carotenoid contents, photosynthetic performance, and P and K uptake; reduced lipid peroxidation (MDA); enhanced antioxidant activity	Chlorophylls, carotenoids, MDA, SOD activity, P and K contents	Si-NPs and S improved drought tolerance by maintaining membrane integrity and nutrient acquisition, reducing oxidative damage and improving plant water status	[151]
Garlic (*Allium sativum* L.)	Field conditions	Elemental sulfur; 0, 15, and 30 L ha^−1^, soil application	Silicon fertilizers (chemical form not specified); 0, 2, and 4 L ha^−1^, soil application	Increased N, P, K, and Mg uptake; improved photosynthetic pigment content; enhanced antioxidant activity; improved nutrient balance	Antioxidant enzyme activities, flavonoids, phenolic compounds, lipid peroxidation (MDA), and N, P, K, and Mg contents	Combined Si and S fertilization enhanced nutrient acquisition and antioxidant capacity while reducing oxidative damage	[162]
Maize (*Zea mays* L.)	Salinity stress (saline soil conditions)	Elemental S, soil application (dose not stated in source)	Potassium silicate, soil application (dose not stated in source)	-	-	Combined application increased grain yield, leaf mineral composition (N, P, K, S, and Si) and grain nitrogen and protein content	[154]
Rice (*Oryza sativa* L.)	Field conditions	Bentonite sulphur; 0, 20, and 40 kg S ha^−1^, basal soil application	Calcium silicate; 0, 150, 300, and 450 kg Si ha^−1^, basal soil application	Increased total tiller number, plant height, grain yield, straw yield, and chlorophyll content	Chlorophyll content	Combined S and Si improved growth and productivity through chlorophyll accumulation and nutrient utilization	[164]
Rice (*Oryza sativa* L.)	Field conditions	Gypsum; 0, 15, 30, and 45 kg S ha^−1^, basal soil application	Fly ash; 0, 40, 80, and 120 kg Si ha^−1^, basal soil application	Increased plant height, tiller number, dry matter production, panicle density, grains per panicle, grain yield, and straw yield	—	Combined S and Si fertilization promoted vegetative growth and yield, likely through improved nutrient availability	[163]
Soybean (*Glycine max* L.)	Field conditions	Sulfur–silicate soluble fertilizer (2.3% S); foliar application at 0, 1.0, 1.33, and 2.0 mL L^−1^ (23, 30.7, and 46 mg S L^−1^), applied six times from 15 to 65 DAP	Sulfur–silicate soluble fertilizer (10% Si); foliar application at 0, 1.0, 1.33, and 2.0 mL L^−1^ (100, 133, and 200 mg Si L^−1^), applied six times from 15 to 65 DAP	Increased branch, leaf, and pod numbers, pod weight, 100-seed weight, plant biomass, and photosynthetic performance	Seed protein content (including the 11S/7S globulin ratio) and reducing sugars	Combined foliar S and Si enhanced pod development and seed quality through improved nutrient utilization	[165]
Winter oat (*Avena sativa* L.)	Severe field drought (June rainfall 6 mm vs. 30-year average of 66.5 mm; +3.2 °C temperature anomaly)	Foliar sulphur fertilization (source and dose not specified)	Potassium silicate, foliar application (dose not specified)	Increased photosynthetic rate, transpiration rate, chlorophyll and carotenoid contents, water-use efficiency, thousand-kernel weight, and grain yield, with reduced leaf area index	Photosynthetic pigments; CO_2_ assimilation, transpiration rate and water use efficiency	Foliar Si and S sustained photosynthetic activity, pigment content and water-use efficiency under drought	[159]
Winter oat (*Avena sativa* L.)	Field conditions (variable weather conditions; no experimentally imposed stress)	Foliar sulphur fertilization (type and dose not specified)	Foliar silicon fertilization (type and dose not specified)	Sulphur increased panicle number, panicle nodes, and spikelets per panicle; silicon increased thousand-kernel weight (TKW); combined Si + S treatment resulted in the highest grain yield, whereas Si alone reduced grain yield	Panicle number, panicle nodes, spikelets per panicle, thousand-kernel weight and grain yield	Foliar Si and S modified yield components through complementary effects on panicle development and kernel weight	[161]
Winter oat (*Avena sativa* L.)	Field drought conditions (2020–2021 growing seasons)	Foliar sulphur fertilization (dose not specified)	Foliar silicon fertilization (dose not specified)	Increased sulphur concentration in shoots and grains; genotype-dependent changes in C and N contents; altered grain protein content; modified macro- and micronutrient accumulation	Carbon (C), nitrogen (N), sulphur (S), protein content, macronutrients (P, K, Ca, Mg, and S), and micronutrients (Al, Ba, Cu, Fe, Mn, Mo, Na, Ni, Pb, Sr, and Zn)	Combined Si and S fertilization altered nutrient uptake and elemental composition, with genotype-dependent changes in S accumulation, protein content and mineral nutrition	[160]

### 4.3. Silicon and Zinc

The integrated application of zinc (Zn) and silicon (Si), particularly in their nanoparticle forms, has proven to be a transformative strategy in modern agronomy. Research indicates that this combination significantly enhances both crop yield and nutritional quality (Table 3). For instance, Kheyri et al. [166] observed that the foliar application of nano-Zn and Si in rice (*Oryza sativa* L.) not only improves grain fortification but also increases protein content more efficiently than traditional methods. Similarly, studies on sage (*Salvia officinalis*) by El-Mahrouk et al. [167] and on rice by Elekhtyar and AL-Huqail [168] suggest that these nanoparticles can drastically reduce the dependency on conventional NPK fertilizers—by up to 75% in some cases—without compromising biomass or essential oil production.

In addition to boosting growth, the Zn-Si pairing serves as a critical defense mechanism against abiotic and biotic stressors. Specifically, when dealing with soil salinity, researchers have found that these elements optimize ionic homeostasis. According to Abd-Elzaher et al. [169] and Shoukat et al. [170], the application of SiO_2_ and ZnO nanoparticles in wheat and maize mitigates salt stress by increasing the potassium-to-sodium (K/Na) ratio, thereby preventing toxic sodium accumulation. Furthermore, this treatment helps maintain osmotic balance and nutrient uptake under saline conditions. Similar protective effects were noted in potato crops by Mahmoud et al. [171], in which the application of ZnCl_2_ and SiCl_4_ enhanced antioxidant enzyme activity to combat oxidative stress.

Moreover, the combination has shown remarkable efficacy in drought management and heavy metal detoxification. For example, Mosaedi et al. [172] and Al-Selwey et al. [173] demonstrated that Si and Zn nanoparticles stabilize relative water content and enhance gas exchange in fennel and potatoes, consequently restoring yields during water deficits. On the other hand, regarding soil contamination, Bakhtiari et al. [174] found that this elemental duo significantly mitigates lead and cadmium toxicity in sage by protecting cell membranes from damage. In addition to the physiological changes seen in various crops, the beneficial effects of the combined application of Si + Zn are closely linked with the regulation of nutrient uptake, ion homeostasis and antioxidant metabolism. Zinc is an important cofactor in a number of enzymes and regulatory proteins that are involved in photosynthesis, protein synthesis, and protecting against oxidative stress. Silicon, in addition to these activities, enhances nutrient-use efficiency, membrane stability and mitigates ionic imbalances under stress. The combined application of Si and Zn significantly increases K^+^ uptake and reduces Na^+^ levels under salinity stress, which helps to maintain the cell metabolism and photosynthetic activity [170]. They also activate the antioxidant defense mechanisms by enhancing the activity of antioxidants, like SOD, CAT, POD and PPO, leading to the enhancement of redox homeostasis and less oxidative damage [171,175,176]. A similar trend was observed in the case of combined nano-fertilizer applications with rice where increased Zn and Si bioavailability, nutrient utilization, protein content, and grain quality were observed [166,168]. Taken together, these results suggest that the beneficial effects of Si and Zn are driven not only by improved plant growth, but also by coordinated regulation of nutrient uptake, ion homeostasis and antioxidant defense mechanisms. Ultimately, whether applied to fruit trees like mango to reduce flower malformation (Elsheery et al. [175]) or to aromatic plants like vetiver to boost essential oils [176], the strategic use of Zn and Si represents a highly versatile and potent tool for sustainable crop intensification.

**Table 3:** Overview of recent studies on the application of zinc (Zn) and silicon (Si) and their effects on agricultural crops.

Plant Species	Stress Condition	Zinc Source (Dose + Application)	Silicon Source (Dose + Application)	Main Physiological Responses	Affected Metabolites/Molecular Responses	Proposed Mechanism	Ref.
Champereia (*Yunnanopilia longistaminea*)	None (greenhouse pot experiment, standard growth conditions)	Nano-ZnO, 30 mg L^−1^, single foliar application; compared with untreated control (no conventional Zn source tested)	Nano-SiO_2_, 30 mg L^−1^, single foliar application; compared with untreated control (no conventional Si source tested)	Both nanoparticles enhanced fresh biomass, plant height, branching, leaf size and chlorophyll (SPAD) content	Increased soluble sugars and proteins; reduced H_2_O_2_, MDA and proline, with lower SOD, POD and CAT activities, indicating reduced oxidative stress	Foliar nanoparticles improved nutrient uptake and metabolic activity while maintaining redox homeostasis	[177]
Maize (*Zea mays* L.)	Salt stress (100 mM NaCl, hydroponic)	Nano-ZnO (100 ppm), Hoagland nutrient solution (hydroponic); compared with conventional ZnSO_4_·7H_2_O	Nano-SiO_2_ (90 ppm), Hoagland nutrient solution (hydroponic); compared with conventional K_2_SiO_3_ at the same concentration	Improved shoot/root fresh and dry biomass, shoot/root length, photosynthetic rate, stomatal conductance, transpiration rate, chlorophyll content (SPAD) and salt tolerance index (STI; nano-Si 120%, nano-Zn 110%, conventional Zn 90%, stressed control 60%); reduced intercellular CO_2_	Nano-Zn enhanced K^+^ uptake and K^+^/Na^+^ ratio, and Zn accumulation in shoots; nano-Si reduced Na^+^ accumulation, enhanced Si accumulation and promoted osmotic adjustment (more negative Ψs)	Nano-Zn acted mainly through K^+^/Na^+^ homeostasis, nano-Si through Na^+^ exclusion and silica deposition	[170]
Mango (*Mangifera indica* L. cv. Ewais)	Salinity stress	Nano-ZnO (50, 100 and 150 mg L^−1^), foliar application at full bloom and one month later, alone or combined with nano-Si	Nano-Si (150 and 300 mg L^−1^), foliar application at full bloom and one month later, alone or combined with nano-ZnO	Improved leaf growth, nutrient status, flowering, fruit yield and fruit quality under salinity; greatest benefits with 100 mg L^−1^ nano-ZnO + 150 mg L^−1^ nano-Si	Increased N, P and K uptake, soluble sugars, total carbohydrates, proline accumulation and antioxidant enzyme activities (SOD, POD, and CAT)	Combined nano-ZnO and nano-Si improved nutrient acquisition, antioxidant defense and osmotic adjustment under salinity	[175]
Potato (*Solanum tuberosum* L.)	Salt-affected sandy soil (EC > 3 dS m^−1^), field conditions	Nano-ZnO; 20 ppm, soil application at 25, 40, 50 and 75 DAP; applied alone (T2) or combined with Si and B (T5), and with Si, B and zeolite (T6); compared with untreated control	Nano-Si; 15 ppm, soil application at 25, 40, 50 and 75 DAP; applied alone (T4) or combined with Zn and B (T5), and with Zn, B and zeolite (T6)	Increased plant height, shoot fresh/dry weight, stem number, relative water content, photosynthetic rate, stomatal conductance, intercellular CO_2_ concentration, water-use efficiency, chlorophyll content, and tuber number; reduced transpiration rate, with the greatest gains under T5 and T6	Leaf N, P, K, Ca, Zn, B and Na contents; proline; GA3; ABA; POD and PPO activities; tuber protein, carbohydrate and starch contents	Combined Zn, Si, B and zeolite improved nutrient uptake, water status, osmotic adjustment (proline), antioxidant defense (POD, PPO) and hormonal balance (decreaseABA, increase GA3)	[171]
Potato (*Solanum tuberosum* L. cv. Hermes)	Drought stress (field, 50/75/100% ETc irrigation)	Nano-ZnO, 50 and 100 ppm, foliar application at 45 and 65 days after planting	Nano-SiO_2_, 25 and 50 ppm, foliar application at 45 and 65 days after planting	Improved photosynthetic performance, stomatal conductance, transpiration, relative leaf water content, chlorophyll content, stomatal integrity, water-use efficiency and tuber yield under drought	Increased photosynthetic pigments and proline accumulation, contributing to osmotic adjustment and improved water status	Nano-ZnO and nano-SiO_2_ maintained photosynthetic activity, water status and stomatal function under drought	[173]
Rice (*Oryza sativa* L.)	Low Si and Zn soil availability	Nano-ZnO, 50 mg L^−1^ (300 g ha^−1^ per spray), foliar application at four growth stages; compared with soil-applied ZnSO_4_ (9 kg ha^−1^)	Nano-SiO_2_, 50 mg L^−1^ (300 g ha^−1^ per spray), foliar application at four growth stages; compared with soil-applied calcium silicate (392 kg ha^−1^)	Increased panicle length, fertile tillers hill^−1^, filled grains panicle^−1^, grain yield and straw yield	Increased Zn and Si concentration/uptake in grain and straw; increased protein content and yield in straw and grain	Improved nutrient availability and uptake, raising Zn/Si accumulation, protein production and yield components; combined nano-Si + nano-Zn gave the largest gain in panicle length	[166]
Rice (*Oryza sativa* L. cv. Giza 179)	None (field study on partial NPK replacement)	Nano-ZnO, 25, 50 and 100 mg L^−1^, foliar application at booting stage, combined with 2/3 of recommended NPK dose	Nano-SiO_2_, 50, 100 and 200 mg L^−1^, foliar application at booting stage, combined with 2/3 of recommended NPK dose	Improved grain yield, yield components, grain quality and nutrient-use efficiency; 50 mg L^−1^ nano-ZnO matched the full NPK dose	Enhanced N, P, K, Zn and Si uptake in grain and straw; increased grain protein content and quality	Nano-ZnO and nano-SiO_2_ improved nutrient uptake and use efficiency, allowing reduced mineral NPK input	[168]
Sage (*Salvia officinalis* L.)	Lead (Pb) and cadmium (Cd) toxicity	Zn nanoparticles (<50 nm), foliar application	Si nanoparticles (<50 nm), foliar application	Increased plant biomass, chlorophyll content and relative water content (RWC); reduced malondialdehyde (MDA) and electrolyte leakage (EL)	Reduced Pb (−40%) and Cd (−36%) accumulation in leaves; increased essential oil yield (+43% with Zn NPs, +37% with Si NPs) and essential oil constituents (1,8-cineole, α-thujone, β-thujone, camphor)	Zn and Si nanoparticles alleviated Pb and Cd toxicity by limiting leaf heavy-metal accumulation, reducing membrane damage and promoting essential oil production	[174]
Sage (*Salvia officinalis* L.)	None (field trial on partial NPK replacement)	Nano-ZnO (1.0 and 1.5 g L^−1^), foliar application (3×), combined with date palm pollen extract (DPE) and reduced NPK	Nano-SiO_2_ (0.1 and 0.2 g L^−1^), foliar application (3×), combined with DPE and reduced NPK fertilization (no conventional Si source tested)	Combined nano-ZnO, nano-SiO_2_ and DPE improved vegetative growth, chlorophyll content, biomass, essential oil yield and partly compensated reduced NPK	Increased K, Zn and Si accumulation; increased total phenolic content, antioxidant activity and essential oil production	Nano-ZnO and nano-SiO_2_ with DPE improved nutrient utilization, antioxidant capacity and secondary metabolism	[167]
Vetiver (*Vetiveria zizanioides* L. Nash)	None (standard cultivation conditions)	ZnO nanoparticles; 50 mg L^−1^, foliar application at 300 DAP	SiO_2_ nanoparticles, 50 mg L^−1^, foliar application at 300 DAP	Increased shoot/root growth, fresh and dry biomass, chlorophyll and carotenoid content, and photosynthetic performance (PSII, carbonic anhydrase activity)	Increased essential oil yield and khusimol content; enhanced antioxidant enzyme activities (SOD, CAT, and POX)	Improved photosynthetic performance and antioxidant defense, raising biomass, essential oil yield and khusimol production	[176]

### 4.4. Silicon and Iron

Modern agricultural research has increasingly focused on the interplay between iron (Fe) and silicon (Si), particularly regarding their ability to shield crops from toxic environments. Instead of relying on single-nutrient applications, recent studies emphasize that the co-application of these elements creates a powerful barrier against heavy metals (Table 4). For instance, work by Ghouri et al. [147] and Yang et al. [178] demonstrates that rice plants grown in hydroponic systems show a remarkable resistance to lead and arsenic when treated with Si and Fe. This is largely because the combination restricts metal translocation to the shoots, thereby reducing arsenic concentrations by over 25% while stabilizing the plant’s physiological state. Similarly, Manzoor et al. [28] found that soil-applied Si and Fe nanoparticles (NPs) protect wheat by stimulating antioxidant enzymes, which consequently prevents toxic elements from reaching the grain.

Beyond just rice and wheat, the Si-Fe partnership has shown exceptional results in neutralizing cadmium (Cd) and chromium (Cr) in various leaf and bean crops. Specifically, when used for seed priming in green and Lima beans, these nanoparticles help restore nutrient balance and boost the production of protective polyamines [179,180]. In the same vein, Irshad et al. [181] reported that treated spinach plants saw a 53% reduction in heavy metal uptake. Moreover, research on maize by Yasin et al. [182] revealed that cadmium accumulation in shoots dropped by a staggering 75%, a result driven by the upregulation of antioxidant genes. Si–Fe interactions have also been linked to iron homeostasis and metal transport processes as well as to stress reduction. One of the important mechanisms is the deposition of iron plaque on the root surfaces that blocks the uptake and movement of toxic elements like arsenic. Application of combined Fe and Si decreased the As content in rice with higher Fe plaque formation and Si competition with As on pathways to aerial tissues, which helped to limit As distribution inside the plants [178]. However, recent studies also suggest that Si and Fe are associated with the regulation of transporter genes related to metal uptake and transport. In the case of combined Fe- and Si-nanoparticle treatments, the exposure to Pb induced the upregulation of Fe- and Si-related transporter genes (*OsIRT2* and *OsLSi1*) and downregulation of the Pb transporter gene (*OsHMA9*), which led to lower accumulation of Pb and higher tolerance to the stress [147]. Moreover, under Cd stress, the combination of Fe and Si decreased the expression of Cd transporters (*ZmNramp5*, *ZmHMA2*, and *ZmHMA3*) and increased the expression of antioxidant genes and the maintenance of cellular redox homeostasis [182]. Together, these results show that the role of Fe-Si interactions goes beyond supplementing Fe and also includes a coordinated regulation of Fe homeostasis, the activity of Fe transporters, Fe sequestration, and metal detoxification pathways.

However, the benefits of this elemental duo extend beyond detoxification; they also significantly enhance growth under climate-related stress. For example, Najafi Disfani et al. [183] observed that iron-silicon nanoparticles accelerate germination and root development in barley and maize far more effectively than standard fertilizers. In addition, Dawa et al. [184] highlighted that foliar sprays of these NPs improve tomato biomass and mineral uptake even during severe water shortages. Likewise, Triticale plants managed to maintain high yields and chlorophyll levels under high salinity by accumulating protective sugars and proline [185].

**Table 4:** Overview of recent studies on the application of iron (Fe) and silicon (Si) and their effects on agricultural crops.

Plant Species	Stress Condition	Iron Source (Dose + Application)	Silicon Source (Dose + Application)	Main Physiological Responses	Affected Metabolites/Molecular Responses	Proposed Mechanism	Ref.
*Common* *bean (Phaseolus vulgaris L.)*	Cd toxicity (CdCl_2_ at 0, 1, 1.5 and 2 mM in pot soil)	Iron oxide nanoparticles (Fe_2_O_3_ NPs), 10 mg L^−1^, applied by seed priming, alone or combined with SiNPs.	Silicon nanoparticles (SiNPs), 20 mg L^−1^, applied by seed priming, alone or combined with Fe_2_O_3_ NPs.	Combined Fe_2_O_3_ and SiNP application improved growth, biomass, leaf relative water content, photosynthetic performance and mineral nutrient status under Cd stress	Increased SOD and CAT activities, polyamines (putrescine and spermidine), nitric oxide (NO), and proline, while reducing MDA and electrolyte leakage, indicating enhanced membrane stability and oxidative stress tolerance.	Combined Fe_2_O_3_ and SiNPs enhanced Cd tolerance through nutrient homeostasis, osmotic adjustment and membrane stability, protecting the photosynthetic apparatus	[179]
*Maize (Zea mays L.)*	Cadmium (Cd) toxicity (Cd-contaminated soil)	Iron nanoparticles (nFe) immobilized on rice husk biochar (B-nFe and B-nSi-nFe); soil amendment	Silicon nanoparticles (nSi) immobilized on rice husk biochar (B-nSi and B-nSi-nFe); soil amendment	The combined B-nSi-nFe treatment improved plant biomass, preserved stomatal morphology and cellular ultrastructure, enhanced soil enzyme activities, and reduced Cd accumulation in roots and shoots.	Increased antioxidant enzyme activities and upregulated antioxidant genes (*ZmAPX*, *ZmCAT*, *ZmPOD*, *ZmSOD*, *ZmGSH*, and *ZmMDHAR*); downregulated Cd transporter genes (*ZmNramp5*, *ZmHMA2*, and *ZmHMA3*); reduced ROS accumulation and bioavailable Cd in soil.	The biochar–nSi–nFe amendment immobilized Cd through Cd–ligand complex formation in the porous biochar matrix, raising soil pH and lowering Cd bioavailability; reduced uptake was associated with suppression of Cd transporter genes and preserved stomatal and cellular integrity	[182]
*Rice (Oryza sativa L.)*	Pb toxicity (100 µM Pb (NO_3_)_2_ in hydroponic culture)	Fe_2_O_3_ nanoparticles, 25 mg L^−1^, applied through the hydroponic nutrient solution, alone or in combined with Si-NPs	SiO_2_ nanoparticles, 2.5 mM, applied through the hydroponic nutrient solution, alone or in combined with Fe-NPs	Combined Fe- and Si-NPs improved growth, biomass and photosynthetic pigment content, preserved root	Enhanced antioxidant enzyme activities (SOD, POD, and CAT), glutathione (GSH), and the expression of antioxidant genes (Os-SOD, OsPOD, and OsCAT), while reducing H_2_O_2_ and MDA levels. Fe- and Si-related transporter genes (*OsIRT2* and *OsLSi1*) were upregulated, whereas the Pb transporter gene (*OsHMA9*) was down-regulated	Combined Fe- and Si-NPs enhanced Pb tolerance by limiting Pb uptake and translocation and regulating metal transporter genes	[147]
*Rice (Oryza sativa L. cv. Shendao 529)*	As toxicity (0.01 mmol L^−1^ Na_2_HAsO_4_ in hydroponic culture)	FeCl_2_, 0.1 mmol L^−1^, applied through the hydroponic nutrient solution alone or combined with Si	H_2_SiO_3_, 1 and 2 mmol L^−1^, applied through the hydroponic nutrient solution alone or combined with Fe	Combined Fe and Si application improved biomass and chlorophyll content while reducing As translocation to shoots, thereby alleviating As toxicity	Reduced MDA and antioxidant enzyme activities (SOD, POD, and CAT), while increasing soluble proteins, soluble sugars, and proline, indicating improved osmotic adjustment	Combined Fe and Si reduced As uptake and shoot translocation through Fe plaque formation and competition between Si and As for transport pathways	[178]
*Spinach (Spinacia oleracea L.)*	Combined Cd and Cr toxicity	Fe_3_O_4_-SiO_2_ nanocomposite, 100 mg L^−1^, applied by seed priming	SiO_2_ incorporated into the Fe_3_O_4_-SiO_2_ nanocomposite	Seed priming with the Fe_3_O_4_–SiO_2_ nanocomposite improved biomass production, leaf area, photosynthetic pigments, gas exchange, and antioxidant capacity under Cd and Cr stress	Increased antioxidant enzyme activities (SOD, CAT, and APX), phenolics, flavonoids, anthocyanins, ascorbic acid, and soluble sugars, while reducing H_2_O_2_, MDA, electrolyte leakage, and Cd/Cr accumulation. Antioxidant genes (SOD, CAT, and APX) were upregulated	The Fe_2_O_3_–SiO_2_ nanocomposite mitigated Cd and Cr toxicity by reducing metal uptake and enhancing antioxidant metabolism and gene expression	[181]
*Tomato (Solanum lycopersicum L.)*	Deficit irrigation (60%, 80% and 100% ETc) under field conditions	Nano-iron, foliar application	Nano-silicon, 12 ppm, foliar application	Deficit irrigation at 80% ETc with nano-Si improved growth, photosynthetic pigments, leaf mineral content, early and total fruit yield and water-use efficiency (WUE)	Increased chlorophyll *a*, chlorophyll *b*, total chlorophyll, leaf N, P, and K contents; increased proline accumulation and WUE under deficit irrigation	Nano-silicon improved photosynthetic capacity, nutrient status and osmotic adjustment (proline) under moderate water deficit	[184]
*Triticale (×Triticosecale Wittm. ex A. Camus)*	Salinity stress (35 and 70 mM NaCl)	Nano-Fe, 1 g L^−1^, foliar application	Nano-Si, 50 mg L^−1^, foliar application; applied alone or in combination with nano-Fe	Combined nano-Fe and nano-Si application improved chlorophyll content, photosynthetic efficiency, relative water content, and grain yield under salinity stress	Increased CAT and POD activities, proline, and soluble sugars, while reducing H_2_O_2_, MDA, and electrolyte leakage	Combined nano-Fe and nano-Si enhanced salinity tolerance through antioxidant defense, osmotic adjustment and membrane stability; further enhanced with PGPR inoculation	[185]
*Wheat (Triticum aestivum L.)*	Arsenic (As) toxicity (42.8 mg As kg^−1^ soil)	Iron oxide nanoparticles (FeONPs), 25, 50 and 100 mg Kg^−1^ soil, applied through irrigation water; tested independently	Silicon oxide nanoparticles (SiONPs), 25, 50 and 100 mg Kg^−1^ soil, applied through irrigation water; tested independently	Both FeONPs and SiONPs alleviated As toxicity by improving plant growth, photosynthetic pigments, and grain yield, while reducing As accumulation in roots, shoots, and grains	Enhanced antioxidant enzyme activities (SOD, POD, and CAT), increased chlorophyll and carotenoid contents, and reduced H_2_O_2_, MDA, electrolyte leakage, and As accumulation	FeONPs and SiONPs independently alleviated As toxicity by limiting As uptake and translocation; FeONPs were more effective, likely through their additional role in Fe nutrition and chlorophyll biosynthesis	[186]

### 4.5. Silicon–Mineral Interactions and the Molecular Regulation of Secondary Metabolism

While phenolic, flavonoid, and terpenoid accumulation are frequently reported as outcomes of Si co-application with Se, S, Zn, and Fe, the molecular mechanisms regulating these responses remain insufficiently explored. These have not prevented recent omics experiments with the individual elements of Se or Si from providing useful clues to the regulatory networks involved in these effects. In case of mini roses suffering from *Podosphaera pannosa*, Si upregulated genes involved in phenylalanine ammonia-lyase, cinnamyl alcohol dehydrogenase and chalcone synthase and increased the accumulation of phenolic acids and flavonoids and thus enhanced disease resistance [187]. Likewise, the transcription factor *OsbZIP18* directly interacted with many phenylpropanoid and flavonoid biosynthesis genes, such as *OsPAL5*, *OsC4H*, *Os4CL*, *OsCHS*, *OsCHIL2*, and *OsF3H*, in rice [188]. Furthermore, when integrating the transcriptomic and metabolomic data, the enrichment of phenylpropanoid biosynthesis pathways were closely related to cold tolerance in tea plants [189]. Taken together, the results imply that Si may cause accumulation of phenolics and flavonoids by the transcriptional reprogramming of pathways related to the phenylpropanoid metabolism.

Combined transcriptomic and proteomic studies showed that selenite and selenate treatments led to upregulation of genes and proteins related to glutathione metabolism and terpenoid-quinone biosynthesis, and effects on the expression of phosphate and sulfate transporters [190]. The results suggest that the uptake of S and Se is closely linked to the process of assimilation, and that their uptake in addition to the changes in redox regulation caused by Si may have a role in magnifying these responses. However, studies specifically focused on combined treatments of Si-Se, Si-S and Si-Zn or Si-Fe are very limited in transcriptomic, metabolomic and proteomic studies.

Interestingly, the molecular regulation of terpenoid and alkaloid biosynthesis upon co-application of Si-mineral has not been studied extensively yet. Other than the reported increase in artemisinin content in Artemisia annua under combined Se-Si treatment, no study found in this review directly studied these pathways by integrated omics approaches. Further study with transcriptomics, metabolomics and proteomics is required to determine if the positive action of Si-mineral interactions on secondary metabolism is related to coordinated regulation of terpenoid- and alkaloid-related biosynthetic genes and pathways.

## 5. Comparative Efficiency, Molecular Basis, and Agronomic Relevance of Silicon Co-Application with Selenium, Sulphur, Zinc, and Iron

Although silicon (Si) co-application with selenium (Se), sulphur (S), zinc (Zn), and iron (Fe) consistently improves plant tolerance to abiotic and biotic stresses, comparative analysis of the reviewed studies indicates that the efficiency and molecular basis of each combination differ according to the nutrient involved, the type of stress, the plant species, and the application strategy, rather than reflecting a universally superior interaction. A comparative summary of the target stress conditions, physiological and molecular mechanisms, recommended application scenarios, agronomic benefits, and interaction patterns of the four Si-based nutrient combinations is presented in Table 5. Si–Se has been examined under the broadest range of constraints, including salinity, drought, heat, heavy-metal toxicity, and biotic stress, and is particularly recommended for metal-contaminated or highly saline environments. Under Cd and Pb stress, Si–Se co-application reduces metal translocation to edible tissues through the repression of metal-transporter genes, including *OsHMA2* and *OsLCT1* [118,144], together with induction of the vacuolar sequestration transporter *OsHMA3* [118,137]. These effects are associated with enhanced glutathione- and phytochelatin-mediated detoxification [118], stimulation of antioxidant defenses, delayed leaf senescence, and improved Se biofortification [143,144,145]. At the metabolic level, this detoxification response is closely associated with modulation of the glutathione- and ascorbate-related antioxidant pathways, most clearly demonstrated for Si–Se co-application in ramie [117], although the relative contribution of Si versus Se to the AsA-GSH cycle appears species-dependent, with Si showing a more limited direct effect in Chinese cabbage compared to Se [139]. For some transporters, however, the regulation of expression is not uniform across studies; for example, *OsNramp5* is repressed in one study [137] and induced in another [118]. Furthermore, several reports showed that combined Si–Se treatments did not always outperform single applications [137,150], indicating that the interaction is strongly dependent on genotype, nutrient form, dose, and environmental conditions.

Compared with Si–Se, Si–S co-application appears particularly beneficial under drought and sulphur-limited conditions. Combined application of Si and S has been reported to improve the osmotic adjustment by uptaking higher levels of osmolytes like proline and soluble sugars and to strengthen the antioxidative defense and photosynthetic activity [151,159,162]. Other improvements include better nutrient assimilation, biomass production, and reproductive ability, leading to higher pod, grain and overall crop production [163,164,165]. The responses indicate that the major role of Si–S is likely more related to enhancing the metabolic homeostasis and nutrient-use efficiency than to direct control of metal-detoxification pathways. Si–Zn combinations work best on saline, droughted or Zn-deficient soils. Zinc is involved in enzymatic activities, nutrient metabolic regulation, and hormone regulation, especially the GA_3_/ABA ratio [171], while Si stimulates stomatal function, membrane stability and osmotic regulation. The combination of these effects leads to more chlorophyll production, higher photosynthetic efficiencies, water-use efficiency and crop productivity under stress conditions [170,173,175]. In maize, enhanced K^+^/Na^+^ homeostasis under salinity conditions has been explicitly reported and under drought conditions, increased relative water content, stomatal integrity and tuber yield have been reported [170,173].

Nevertheless, under conditions of adequate nutrient supply, several responses appear mainly additive rather than truly synergistic, especially regarding nutrient accumulation and yield-related traits [166]. Among the four combinations, Si–Fe exhibits one of the strongest mechanistic bases under heavy-metal stress. Studies conducted under Cd, Pb, As, and Cr toxicity showed that Fe–Si co-application reduces toxic-metal uptake through Fe-plaque formation and coordinated regulation of Fe- and Si-related transport systems, including *OsIRT2* and OsLsi1 [147,178]. These processes are accompanied by improved antioxidant activity, maintenance of Fe homeostasis, preservation of photosynthetic function, and reduced oxidative damage [147,178,179,181,182].

In particular, combined Fe–Si treatments were specifically reported as synergistic under Cd and Pb toxicity [147,179] and nanocomposites and biochar-based treatments also showed coordinated regulation of antioxidant metabolism, transporter genes and metal immobilization mechanisms [181,182].

In all four nutrient combinations, there are several common responses that can be noted such as the strengthening of antioxidant enzyme systems (SOD, CAT, POD, and APX), supported at the transcriptional level by upregulation of the corresponding gene (*OsSOD*, *OsPOD*, and *OsCAT* [147]; *ZmAPX*, *ZmCAT*, *ZmPOD*, and *ZmSOD* [182]) stabilization of cellular membranes, maintenance of photosynthetic activity, and maintenance of ion and nutrient homeostasis. However, the main target mechanisms vary significantly between nutrients as follows: Si–Se is mainly used to regulate metal transport and detoxification processes, Si–Zn mainly affects ionic balance and hormonal regulation, and Si–S mainly supports osmotic adjustment and acquisition of nutrient/metabolic stability. Species-specific metabolic responses have also been reported, such as activation of the proline–P5C–GABA pathway under Si–Se treatment in fragrant rice [142], illustrating the diversity of metabolic networks engaged by these interactions. It should be noted that molecular evidence for Si–S and Si–Zn interactions remains comparatively less developed at the transporter and signaling levels than for Si–Se and Si–Fe, reflecting a genuine gap in the current literature rather than a limitation of this review.

**Figure 4:**
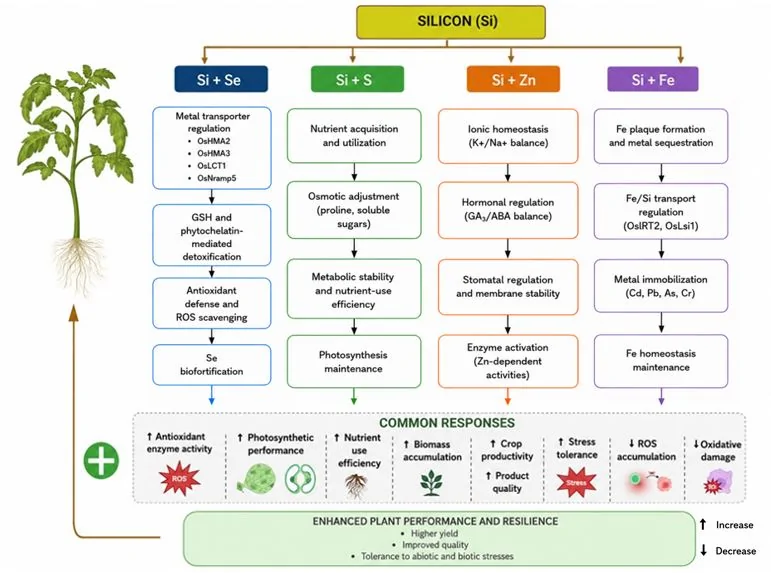
Schematic representation of the comparative mechanisms, optimal applications, and major benefits of silicon (Si) co-application with selenium (Se), sulphur (S), zinc (Zn), and iron (Fe) for improving plant resilience under stress conditions (created with BioRender.com).

Synergy is therefore not universal and is greatly influenced by plant species, stress conditions, and application strategy. Synergistic interactions have been reported only for Si–Se and Si–Fe under heavy-metal stress [118,179], and the evidence is limited: neither study tested the Si × element interaction term, and the claim rests on mean separation between treatments. In contrast, many other reported benefits are more appropriately classified as additive, species-dependent, or condition-specific responses. A conceptual framework summarizing the comparative efficiency, dominant mechanisms, and agronomic relevance of the four Si-based nutrient combinations is presented in Figure 4.

## 6. Conclusions and Perspectives

Mineral elements play a key role in plant growth, development, and productivity and participate in the regulation of essential processes including photosynthesis, enzyme activation, osmotic adjustment, redox homeostasis, protein synthesis, and secondary metabolism. However, under abiotic stresses such as drought, salinity, heavy metal stress, and nutrient imbalance, the uptake, translocation, and homeostasis of these elements are greatly disrupted, negatively affecting crop performance and nutritional quality.

This review focused on consolidating current knowledge on the co-application of silicon (Si) with the selected mineral elements selenium (Se), sulphur (S), zinc (Zn), and iron (Fe) and their effects on plant physiological responses, stress tolerance mechanisms, nutrient homeostasis, and modulation of secondary metabolism. Si, though not essential for most higher plants, has consistently been shown to have a positive effect in sustainable crop production under both optimal and adverse environmental conditions. The reviewed literature indicates that co-application of Si with Se, S, Zn, and Fe can produce beneficial, often complementary or additive responses, whereas true synergistic interactions remain insufficiently demonstrated. This includes the enhancement of antioxidant defense systems, regulation of reactive oxygen species (ROS) homeostasis, improved nutrient uptake and translocation, stabilization of photosynthetic activity, improvement of ion and water homeostasis and modulation of primary and secondary metabolic pathways such as biosynthesis of phenolic compounds, flavonoids and sulphur-containing metabolites, accumulation of amino acids in response to Si–Se and Si–S interactions, and enhancement of terpenoid secondary metabolites in selected species. The Si and Se combination is particularly promising in the context of antioxidant protection, heavy metal stress mitigation and biofortification of crops. Co-application of Si and S helps facilitate the synthesis of sulphur-containing compounds, protein synthesis and drought and salinity tolerance. Si and Zn combination has been found to be effective under various stress conditions for ionic balance, chlorophyll content, yield, and nutrient accumulation. In turn, Si and Fe interactions reduce the toxic metal adsorption, especially Cd, Pb, As, and Cr, and enhance antioxidant activity and productivity of plants. In general, co-application of Si with mineral elements is a promising and scientifically supported approach to enhance crop resilience, efficiency of mineral element uses and biofortification potential. Further research is needed on its integration into sustainable agricultural systems, especially in the face of growing environmental challenges posed by climate change.

Although the reviewed literature indicates significant progress, several basic questions remain unresolved and require targeted investigation in future studies. Si uptake is mainly controlled by Lsi1 and Lsi2, which are influx and efflux transporters, respectively, whereas *Lsi3* and *Lsi6* are involved in xylem loading, unloading, and internal Si redistribution, especially in rice [12,62]. It is not known, however, how these Si transport systems interact with transporters of mineral nutrients, or whether Se, S, Zn, and/or Fe affect the uptake kinetics, xylem loading, or deposition of Si in tissues. This is an important gap in knowledge since it has been demonstrated that Si impacts nutrient availability, acquisition, uptake, and translocation in plants, but the molecular mechanisms remain unclear [23]. Additionally, future research should focus on the expression of salt stress and ion transport associated genes (*SOS1*, *NHX*, and *HKT*) in response to combined Si mineral treatments and stress conditions, as they are key to Na^+^/K^+^ homeostasis and salt stress adaptation [191,192]. Identification of molecular targets of Si mineral interactions and metabolic pathways most sensitive to these co-applications would be necessary to understand how these elements interact in plants. For this purpose, integrated approaches such as transcriptomics, proteomics, metabolomics, and ionomics will be required [193,194]. Water relations are another understudied aspect. Aquaporins have been reported to regulate plant hydraulic conductance, stomatal conductance, and transpiration [52]; Si has been reported to improve the water status of plants through upregulation of aquaporin gene expression and an increase in hydraulic conductance in the root under osmotic and salt stress [89,95,193]. The potential modulation of aquaporin-related gene expression to further enhance water-use efficiency in plants under combined Si–element treatments and stress conditions needs to be systematically investigated, especially under combined drought-salinity stress.

The form, timing, and mode of application of both Si and the co-applied mineral elements have a major effect on treatment efficacy. A wide range of Si sources and formulations has been tested, with very different results depending on crop species, soil type, developmental stage, application technique, and stress intensity [21,40,42]. Further dose response and comparative application studies are required to determine the optimum combinations and concentrations for different cropping systems such as soil application, foliar application, fertigation, hydroponics, and seed priming [15,39,195].

New opportunities in sustainable crop production with the use of nanoparticle-based formulations, such as nano-Si, nano-Se, nano-Zn and nano-Fe, exist but come with significant concerns. It is important to critically acknowledge that a large proportion of the evidence discussed throughout this review, particularly regarding Si–Se and Si–Fe co-application under heavy-metal stress, originates from hydroponic, pot-based, or greenhouse experiments, and a substantial share relies on nanoparticle formulations rather than conventional soluble sources [113,140,150]. While these controlled systems have been instrumental in elucidating the physiological and molecular mechanisms underlying Si-nutrient interactions, they do not reproduce the soil heterogeneity, microbial activity, nutrient competition, and climatic variability encountered under field conditions. The feasibility of translating these findings into practical agricultural recommendations is therefore not yet established, and the additional cost, regulatory complexity, and variable efficacy associated with nanoparticle-based fertilizers including hetero-aggregation effects that have already been reported to reduce treatment efficiency [140,150] further limit their immediate applicability at field scale, underscoring the need for comprehensive life-cycle and cost–benefit assessments before large-scale adoption [166,167,168]. Their long-term persistence in soil ecosystems remains insufficiently characterized in soil ecosystems, with impacts on microbial diversity, bioaccumulation in edible parts, and other ecotoxicological impacts [196]. For large-scale use of Si and mineral fertilizers based on nanoparticles to be considered responsibly, comprehensive life cycle assessments and regulations should be developed first [197,198]. Some of the most promising but still underexplored areas are the co-application of Si with mineral elements, especially their role in regulating secondary metabolism and enhancing the potential of biofortification. Further research should systematically analyze the profile of secondary metabolites (phenolics, flavonoids, organosulphur compounds, and terpenoids) in response to the interaction between Si and mineral treatments, particularly in field-grown crops. For biofortification strategies suitable for human nutrition and public health, nutrient speciation, bioavailability, and safe accumulation limits should be assessed for Se, Zn, and Fe [25,35]. Building on the knowledge gaps identified above, we highlight several interconnected priorities that should guide future research in this field. First, systematic investigation of nutrient transporter regulation is needed to clarify whether Si transport systems (Lsi1, Lsi2, Lsi3, and Lsi6) interact directly or indirectly with Se-, S-, Zn-, and Fe-related transporters, and how such interactions are regulated at the transcriptional and post-translational levels [12,23,62]. Second, molecular breeding approaches should be explored to determine whether selection for favorable alleles of Si- and nutrient-transporter genes could reduce the pronounced genotype-dependent variability observed in several Si–Se and Si–Zn studies, thereby improving the consistency of co-application benefits across cultivars and species. Third, the role of rhizosphere and endophytic microbiome interactions in modulating the efficiency of Si-nutrient co-application remains largely unexplored, despite evidence that nanoparticle-based fertilizers can influence soil microbial communities [166]; clarifying these plant–microbiome interactions could reveal additional pathways through which Si and co-applied elements affect nutrient acquisition and stress tolerance. Fourth, precision nutrient management strategies integrating soil and plant diagnostics, remote sensing, and decision-support tools to optimize the dose, timing, ratio, and application method of Si and co-applied nutrients for specific crop–soil–climate combinations will be essential to move from generalized recommendations toward site-specific fertilization practices. Finally, and most critically, large-scale, multi-season, multi-location field validation, combined with the integrated omics approaches discussed above [163,165], will be indispensable to confirm whether the synergistic effects reported under controlled conditions translate into reproducible, economically viable, and environmentally safe outcomes under real agricultural production systems.

To conclude, most of the reviewed studies were conducted under controlled conditions, such as hydroponic systems, pots, or greenhouses. These studies mainly focused on the following Si-accumulating cereal crops: rice, wheat, maize, and barley [39,199,200]. However, the responses of dicot crops, aromatic plants, and perennial fruit trees remain poorly studied under field conditions. Evidence from perennial crops, including olive, indicates that Si–element interactions may influence phenolic profiles and amino acid composition, but broader field validation across species and environments is still required [128]. There is a critical need for additional long-term field trials in various climatic and soil conditions, stress conditions, and agronomic management systems, including physiological, biochemical, molecular, agronomic, economic, and environmental evaluations [40,42]. Integrated research is needed to determine whether the positive effects observed under controlled conditions can be confirmed under field conditions and to establish practical and crop-specific guidelines for using Si in combination with Se, S, Zn, and Fe as a combined agronomic strategy. Overall, the combined application of Si with selected essential and beneficial elements may represent a promising strategy to improve crop resilience, nutrient use efficiency, and nutritional quality, particularly under the growing challenges of climate change and increasing food demand.

## Figures and Tables

**Table 1:** Overview of recent studies on the application of selenium (Se) and silicon (Si) and their effects on agricultural crops.

Plant Species	Stress Condition	Selenium Source (Dose + Application)	Silicon Source (Dose + Application)	Main Physiological Responses	Affected Metabolites/ Molecular Responses	Proposed Mechanism	Ref.
Brown rice (*Oryza sativa* L. ‘Changliangyou 772’)	Cadmium stress in slightly Cd-contaminated acidic (pH 4.77) and calcareous purple (pH 7.77) paddy soils	Se-containing Si fertilizer (L-SiSe), foliar spray (dose not reported)	Silicon fertilizer as soil amendment (S-Si), foliar spray of Si alone (L-Si), or foliar spray of Se-containing Si (L-SiSe)	Increased yield in calcareous soil; reduced grain Cd (up to 50%); increased protein and Se content	Downregulation of Cd transporter genes	Soil Si raises pH and immobilizes Cd; foliar Si forms Si–Cd complexes in stems and leaves, limiting translocation; Se enhances Cd retention in roots	[138]
Chinese cabbage (*Brassica campestris* L. ssp. chinensis var. utilis)	Cadmium stress (0, 1, 5 mmol L^−1^ CdCl_2_, hydroponic culture)	Sodium selenite (Na_2_SeO_3_), 0, 1, 5 mmol L^−1^, supplied in the nutrient solution	Sodium silicate (Na_2_SiO_3_), 0, 1, 5 mmol L^−1^, supplied in the nutrient solution	Increased shoot and root biomass; decreased MDA and H_2_O_2_ levels; enhanced SOD, CAT, and APX activities	Increased glutathione (GSH) and ascorbate (AsA); enhanced GR and DHAR activities, particularly with Se; enhanced SOD, CAT, and APX with both Se and Si	Se enhanced the antioxidant system and stimulated the GSH–AsA cycle; Si showed limited effect on the cycle, suggesting a separate protective mechanism	[139]
Common bean (*Phaseolus vulgaris* L.)	Leaf spot disease caused by *Alternaria alternata*	Biological nano-selenium (nano-Se; 100 ppm), foliar spray	Nano-silica (nano-SiO_2_; 200 ppm), foliar spray; combined treatment: nano-Se (50 ppm) + nano-SiO_2_ (100 ppm), foliar spray	Increased stem length, leaf number, biomass, chlorophyll a/b, Fv/Fm, CAT, POX, PPO, seed yield and disease resistance; reduced disease severity	Enhanced CAT, POX, and PPO activities; increased total antioxidant content; reduced electrolyte leakage	Nano-Se and nano-SiO_2_ reduced oxidative damage and increased resistance to A. alternata; combined application further improved plant immunity, although hetero-aggregation may have reduced efficiency	[140]
Date palm (*Phoenix dactylifera* L. cv. Zaghloul)	Non-stressed field conditions	Sodium selenite (Na_2_SeO_3_·5H_2_O), 0.01% or 0.02%, foliar spray, four times during the growing season	Potassium silicate (25% Si + 10% K_2_O), 0.05% or 0.1%, foliar spray, four times, alone or combined with Se	Combined Si–Se enhanced leaf area, chlorophyll, leaf N/P/K, bunch weight, fruit yield/size, pulp percentage, and pulp-to-seed ratio; improved TSS and sugars; decreased acidity, seed%, tannins, and crude fiber	Increased leaf N, P, K, chlorophyll, TSS, and total/reducing sugars; reduced tannins and crude fiber	Combined Si–Se application improved nutrient uptake and photosynthetic capacity; Si enhances water and mineral acquisition; Se strengthens antioxidant metabolism and carbohydrate biosynthesis	[141]
Fragrant rice (*Oryza sativa* L. cv. Xiangyaxiangzhan and Yuxiangyouzhan)	Normal pot and field cultivation	Selenium ore powder (80 mg kg^−1^ Se); pot: 0, 120, 240 mg kg^−1^ soil; field: 0, 300, 600 kg ha^−1^; soil application before transplanting	Water-soluble silicon fertilizer (SiO_2_ ≥ 72%); pot: 0 or 60 mg kg^−1^ soil; field: 0 or 150 kg ha^−1^; soil application before transplanting	Increased grain yield, head rice yield, dry biomass, and leaf area index; enhanced SOD, POD, CAT; reduced MDA; increased 2-acetyl-1-pyrroline (2AP); improved grain quality; reduced lodging risk with Si	Increased proline, pyrroline-5-carboxylate (P5C), and γ-aminobutyric acid (GABA); positive correlations between SOD activity and grain yield, and between 2AP content and grain quality	Combined Se and Si improved growth, antioxidant capacity, grain quality, and aroma via proline–P5C–GABA metabolism and reduced oxidative damage; Si improved stem mechanical strength, reducing lodging	[142]
Lentil (*Lens culinaris* Medik.)	Heat stress (35/25 °C)	Sodium selenate; soil drench before heat stress, than foliar spray 7 days after (1–10 μM; effective: 2.5 and 5 μM)	Sodium silicate; soil drench before heat stress, followed by foliar spray 7 days after (1–10 μM; effective: 2.5 and 5 μM)	Improved shoot and root growth, leaf water status, chlorophyll content, and photosynthetic performance; reduced membrane damage and oxidative stress	Increased APX and GR activities; increased AsA, reduced GSH; reduced MDA and H_2_O_2_	Combined Se–Si application enhanced heat tolerance by strengthening antioxidant defense and improving redox homeostasis	[114]
Maize (*Zea mays* L.)	Salt stress (150 mM NaCl)	Sodium selenite (dose not specified), applied in combination with Si	Silicon (1 mM, chemical form not specified), seed priming before salt stress, combined with Se	Improved root and shoot growth; preserved photosynthetic performance; enhanced SOD and CAT activities	Upregulated salt-tolerance genes (*ZmNHX*, *ZmSOS1*, *ZmSOS2*, and *ZmHKT1*); increased net Na^+^ efflux; reduced root Na^+^ accumulation	Si–Se co-application restricted Na^+^ uptake and translocation via ion transporter regulation, promoting Na^+^ exclusion and vacuolar compartmentalization	[115]
Oregano (*Origanum vulgare* L.)	Drought stress (30%, 70%, and 100% field capacity)	Sodium selenite, foliar spray (15 and 30 mg L^−1^; optimum 30 mg L^−1^)	Silicic acid, foliar spray (150 and 300 mg L^−1^; optimum 150 mg L^−1^)	Improved shoot fresh and dry biomass, enhanced drought tolerance, and increased photosynthetic performance	Increased proline, soluble sugars, chlorophylls, carotenoids, and total phenolic compounds	Se–Si co-application enhanced osmotic adjustment, preserved the photosynthetic apparatus and maintained plant water status	[116]
Ramie (*Boehmeria nivea* L. ‘Gaud’)	Cadmium stress (5 mg L^−1^ Cd, hydroponic culture)	Sodium selenite (1 μmol L^−1^), hydroponic application	Sodium silicate (1 mmol L^−1^), hydroponic application	Increased biomass and chlorophyll; reduced Cd accumulation and root-to-shoot translocation; enhanced SOD, POD, APX, and GR; decreased H_2_O_2_ and MDA	Increased GSH and AsA, modulation of vitamin E, enhanced antioxidant defense via the AsA–GSH cycle	Se–Si co-application alleviated Cd toxicity by limiting Cd uptake/translocation, enhancing antioxidant defenses via the AsA–GSH cycle, and promoting Cd detoxification and sequestration	[117]
Rice (*Oryza sativa* L.)	Salinity stress (saline soil)	Selenium nanoparticles (Se-NPs, 6.25 mg L^−1^), grain soaking (overnight) and foliar spray at maximum tillering (MT), panicle initiation (PI), and booting (BT) stages	Silicon nanoparticles (Si-NPs, 12.5 mg L^−1^), grain soaking (overnight) and foliar spray at MT, PI, and BT stages	Increased root thickness and volume, relative water content, dry matter, leaf area index, and grain yield; improved salt tolerance	Enhanced antioxidant activity and ion homeostasis through reduced Na^+^ accumulation, increased K^+^ uptake, and a lower Na^+^/K^+^ ratio	Se–Si nanoparticle co-application maintained Na^+^/K^+^ homeostasis, reduced Na^+^ uptake, preserved photosynthetic activity and delayed leaf senescence	[143]
Rice (*Oryza sativa* L. subsp. indica cv. Jiuliangyou Huanglizhan)	Combined Cd and Pb stress (3.0 mg kg^−1^ Cd + 300 mg kg^−1^ Pb, contaminated pot soil)	Selenium nanoparticles (Se-NPs; 4, 6, 12 μg mL^−1^), foliar application as a Si–Se nanocomposite, four times from tillering to early booting	Silica nanoparticles (Si-NPs; 15, 22, 44 μg mL^−1^), foliar application as a Si–Se nanocomposite, four times from tillering to early booting	Si–Se nanocomposite reduced Cd and Pb accumulation in leaves and brown rice, slightly improved grain yield, enhanced Pn, Gs, and Tr, and decreased intercellular CO_2_ concentration	Downregulated Cd transport genes (OsHMA2, OsPCR1, OsLCT1, OsCCX2, and TaCNR2), increased selenium-binding protein 1 (SBP1); increased	Si–Se nanocomposites reduce Cd/Pb translocation to grains by repressing metal transporter genes and enhancing SBP1-mediated sequestration	[144]
Rice (*Oryza sativa* L. cv. WYHZ, NJ5055, ZF1Y)	Cadmium-contaminated paddy soil (total Cd = 0.82 mg kg^−1^; pH 5.53)	Sodium selenite (Na_2_SeO_3_), 40 mg L^−1^ Se + 0.1% Tween 80, foliar spray at tillering and heading stages	Silicon nanoparticles synthesized from tetraethyl orthosilicate (TEOS), 2.5 mM Si + 0.1% Tween 80, foliar spray at tillering and heading stages	In cv. WYHZ, combined Si–Se treatment increased Pn, Gs, and Tr; Cd accumulation markedly decreased in brown rice, roots, and stems, with no significant improvement in NJ5055 and ZF1Y	Reduced root-to-stem and stem-to-grain Cd translocation, increased stem-to-leaf translocation; stem Cd concentration was the main determinant of grain Cd; repression of OsLCT1 and OsNramp5, induction of OsHMA3	Si deposition as silica beneath the cuticle forms a physical barrier restricting Cd transport, particularly at stem nodes; Se alleviates Cd-induced oxidative stress; efficacy influenced by nanoparticle size (Si > Se > Si + Se)	[137]
Rice (*Oryza sativa* L. cv. Youyu-8050)	Cd- and Pb-contaminated paddy field (soil Cd 0.84–0.85 mg kg^−1^; Pb 86.12–106.46 mg kg^−1^)	Selenium nanoparticles (Se-NPs; 5, 10, 20 mg L^−1^), foliar application at late tillering and heading stages	Silicon nanoparticles (Si-NPs; 5, 10, 20 mg L^−1^), foliar application alone or combined with Se-NPs at the same stages	Combined Se–Si nanoparticles increased grain yield and biomass, reduced Cd/Pb below the Chinese safety limit, enhanced grain Se biofortification and protein content, decreased phytic acid; Se_3_Si_2_ treatment gave the greatest improvement	Organic Se 74% of total grain Se (protein-, starch-, and lipid-bound); reduced root-to-shoot Cd translocation; strong negative correlations between grain Se and Cd, Pb, phytic acid, and positive with yield and biomass	Combined Si and Se nanoparticles mitigate Cd/Pb accumulation through Si-mediated metal complexation, cell wall and cuticular barriers, and regulation of Cd transporter genes	[145]
Rice (*Oryza sativa* L. cv. EZhong 5)	Cadmium stress (100 μM CdCl_2_·2H_2_O, hydroponic culture)	Sodium selenite (Na_2_SeO_3_), 3 μM, supplied through the nutrient solution	Sodium silicate (Na_2_SiO_3_·9H_2_O), 1.5 mM, supplied through the nutrient solution	Combined Si–Se restored plant height, root length, biomass, Pn, Gs, Tr, and chlorophyll versus Cd-stressed plants; combined treatment showed the greatest mitigation of Cd toxicity	MDA decreased; GSH and phytochelatin (PC) increased; reduced Cd translocation via root cell wall/vacuolar sequestration; downregulation of *OsHMA2* and upregulation of *OsNramp1*, *OsHMA3*, and *OsNramp5*	Combined Si–Se application alleviated Cd toxicity by restricting root-to-shoot Cd transport, enhancing GSH-/PC-mediated chelation and promoting vacuolar sequestration	[118]
Rice (*Oryza sativa* L. cv. Jingzao 47)	Cadmium stress in Cd-contaminated paddy soil (0.82 mg Cd kg^−1^ soil)	Sodium selenite (Na_2_SeO_3_), 40 mg L^−1^, foliar spray (100 mL pot^−1^) at tillering and heading stages	Tetraethyl orthosilicate (TEOS, C_8_H_20_O_4_Si), 2.5 mmol L^−1^, foliar spray (100 mL pot^−1^); sodium sulfide biofuel ash (SSBA) or lime applied to soil at 0.1% (*w*/*w*)	Increased yield (4–10%), enhanced antioxidant defense, and reduced brown-rice Cd accumulation by up to 83%, particularly with combined Se and Si foliar application	Increased CAT and POD activities; enhanced grain Se accumulation; reduced CaCl_2_-extractable (bioavailable) soil Cd following SSBA or lime application	Soil amendments immobilized Cd by raising soil pH; foliar Se and Si restricted Cd uptake and translocation via complexation and cell-wall sequestration; no significant synergy between soil amendments and foliar Se–Si	[146]
Rice (*Oryza sativa* L. cv. Basmati-515)	Drought stress (40% water holding capacity, pot experiment)	Selenium (chemical form not specified), 0.5 mM, foliar spray (10 mL pot^−1^), every 10 days from drought onset	Silicon (chemical form not specified), 1.5 mM, foliar spray (10 mL pot^−1^), every 10 days from drought onset	Combined Si–Se restored shoot length, biomass, RWC, chlorophyll (SPAD), and grain yield to near control levels	—	Combined Si–Se application alleviated drought-induced growth inhibition by improving water status and preserving photosynthetic capacity	[147]
Strawberry (*Fragaria × ananassa* Duch.)	Drought stress (30%, 60%, and 100% field capacity)	Selenium nanoparticles (Se-NPs), foliar spray (25 mg L^−1^), applied three times weekly	Silicon dioxide nanoparticles (SiO_2_-NPs), foliar spray (125 mg L^−1^); combined Se/SiO_2_-NPs (50 and 100 mg L^−1^)	Improved root and shoot biomass, photosynthetic pigments, chlorophyll fluorescence, RWC, membrane stability index, water-use efficiency and antioxidant enzymes (SOD, CAT, APX, and GPX); reduced MDA and H_2_O_2_	Increased osmolytes (proline, soluble carbohydrates) and fruit bioactive compounds (anthocyanins, total phenolics, and vitamin C); enhanced antioxidant capacity (DPPH assay)	Se–SiO_2_ nanoparticle co-application promoted osmotic adjustment and stimulated antioxidant metabolite accumulation, improving fruit quality	[113]
Sweet wormwood (*Artemisia annua* L.)	Non-stressed field conditions	Sodium selenate, 50 mg L^−1^, foliar application twice (mid-June and early August), alone or combined with Si-NPs	Silicon nanoparticles (13–14 mg L^−1^), produced by pulsed laser ablation, foliar application twice, alone or combined with Se	Se and/or Si stimulated vegetative growth; Se increased chlorophyll a, Si alone reduced it; ascorbic acid and total antioxidant activity increased with individual application; combined Se–Si increased artemisinin, though essential oil yield decreased	Se and Si altered terpenoid profiles; combined treatment increased artemisinin and artemisia ketone, reduced camphor; antioxidant activity correlated with polyphenols; Se accumulated preferentially in leaves; combined Se–Si reduced Cd, As, Pb, Sr, Cr, Ni, and V accumulation	Se enhances chlorophyll biosynthesis and terpenoid metabolism, and Si promotes glandular trichome development for artemisinin biosynthesis; combined application improves artemisinin production and Se biofortification and reduces heavy-metal accumulation	[148]
Tomato (*Solanum lycopersicum* L.)	Salt stress (150 mM NaCl)	Sodium selenate (Na_2_SeO_3_, 10 µm), applied either by root irrigation or foliar spray	Sodium silicate Na_2_SiO_3_, 2 mM), applied either by root irrigation or foliar spray	Improved fresh weight and root length, chlorophyll content and photosynthetic performance (Pn, Gs, Tr, and Fv/Fm); reduced electrolyte leakage, H_2_O_2_ and MDA; enhanced K^+^ uptake and reduced Na^+^ accumulation	Enhanced antioxidant enzyme activities (SOD, CAT, POD, DHAR, and GR), reduced oxidative damage (H_2_O_2_ and MDA), regulation of ion transporter genes (SOS1, HKT1, and NHX1), improved Na^+^/K^+^ homeostasis	Si improved ionic homeostasis, Se strengthened antioxidant defense; combined application enhanced salt tolerance—coordinated regulation of photosynthesis, antioxidant metabolism and Na^+^/K^+^ balance, foliar treatment favoring photosynthesis and root application ionic homeostasis	[149]
Tomato (*Solanum lycopersicum* L. cv. Chilian 1)	Non-stressed greenhouse conditions	Sodium selenite (Na_2_SeO_3_), 25 μM, foliar spray applied twice during fruit set/enlargement	Sodium silicate (Na_2_SiO_3_), 1.0 mM, foliar spray applied twice, alone or combined with Se	Si and Se improved vegetative growth; combined treatment further enhanced plant height, stem diameter, and leaf number; fruit yield/number increased with Si and Si + Se; fruit weight unchanged	Increased soluble sugars, vitamin C, phenolics, anthocyanins, lycopene, carotenoids, and soluble proteins, Si increased sucrose and soluble proteins, decreased nitrate; combined treatment enhanced flavonoids	Si primarily promotes vegetative growth and fruit yield, Se enhances nutritional quality; combined application is mainly additive rather than synergistic, indicating species- and cultivar-dependent interactions	[136]
Wheat (*Triticum aestivum* L. var. Puxing 5)	Cadmium stress (Cd-contaminated soil)	Nano-Se (dose not specified), soil amendment, foliar spray, seed soaking, or seed coating	Nano-Si (dose not specified), soil amendment, foliar spray, seed soaking, or seed coating	Improved plant growth and grain yield (up to 25.83%); markedly reduced grain Cd accumulation (31.30–62.99% with nano-Se; 11.39–51.04% with nano-Si)	Downregulated *TaNramp5* and *TaLCT1*, upregulated *TaHMA3*; enhanced SOD and POD activities; reduced MDA accumulation	Nano-Se reduced Cd bioavailability and promoted root immobilization, while nano-Si enhanced cell-wall binding; their combination formed aggregates that reduced uptake efficiency	[150]
Wheat (*Triticum aestivum* L. cv. JB Asano)	Cadmium stress (10 μM CdCl_2_, hydroponic culture)	Sodium selenite (Na_2_SeO_3_), 1.5 μM, foliar application	Sodium silicate (Na_2_SiO_3_), 0.2 mM, supplied through the nutrient solution	Cd reduced biomass, shoot length, tiller number, chlorophyll, photosynthetic rate and transpiration; combined Se–Si gave the greatest improvement and Cd reduction	Reduced ROS, H_2_O_2_, MDA, and electrolyte leakage; increased total protein; enhanced CAT, APX, GR, and GSH; decreased GSSG; Cd negatively correlated with Se and Si	Si and Se confer complementary protection through an avoidance–tolerance strategy: Si limits Cd uptake and translocation via cell-wall immobilization and apoplastic barriers, Se enhances GSH-mediated detoxification	[28]
Wheat (*Triticum aestivum* L. cv. Faisalabad-2008)	Salinity stress (50 mM NaCl, pot experiment)	Selenium (chemical form not specified), 40 mM, foliar spray applied once 7 days after salt stress	Silicon (chemical form not specified), 40 mM, foliar spray applied once every 7 days after salt stress	Alleviated salt-induced reductions in biomass, RWC, leaf water potential, chlorophyll, Pn, gs, transpiration, and osmotic adjustment; Si alone also improved biomass and root-to-shoot ratio	Enhanced CAT, SOD, POD, and nitrate reductase; increased proline, soluble proteins, and soluble sugars	Combined Si–Se application enhanced salinity tolerance via improved water relations and osmotic adjustment; Si contributes structural protection; Se enhances antioxidant and N metabolism	[29]
Wheat (*Triticum aestivum* L. cv. Faisalabad-2008)	Drought stress (40% water holding capacity, pot experiment)	Selenium (chemical form not specified), 40 mM, foliar spray (10 mL pot^−1^), 7 days after drought induction	Silicon (chemical form not specified), 40 mM, foliar spray (10 mL pot^−1^), alone or combined with Se, 7 days after drought induction	Combined Si–Se alleviated drought-induced reductions in growth, biomass, RWC, water/turgor potentials, chlorophyll, Pn, Tr, gs, and intercellular CO_2_	Enhanced SOD, POD, and CAT, positively associated with osmotic adjustment and turgor maintenance	Combined Si–Se application enhanced drought tolerance by improving water status, reinforcing structural barriers and preserving chloroplast function	[119]
Wheat (*Triticum aestivum* L. cv. Misr3)	Salinity stress (field conditions; soil EC 7.61 dS m^−1^)	Sodium selenate (Na_2_SeO_4_), 15 or 30 mM, foliar spray at BBCH 13 and BBCH 23	Sodium silicate (Na_2_SiO_3_), 15 or 30 mM, foliar spray at BBCH 13 and BBCH 23, alone or combined with Se	Combined Si–Se improved plant height, tiller number, biomass, Fv/Fm, performance index, membrane stability index, RWC, anatomical traits and yield components; Se30 + Si30 gave the greatest improvement	Enhanced osmolyte accumulation (soluble sugars and proline), non-enzymatic antioxidants (GSH and AsA), and antioxidant enzymes (SOD, CAT, APX, and GR)	Combined Si–Se application enhanced salinity tolerance by reinforcing structural and vascular tissues, improving water retention and nutrient transport	[30]

**Table 5:** Comparative characteristics of Si–Se, Si–S, Si–Zn and Si–Fe co-application in plants: the evidence base compiled in this review.

Combination	Target Stresses and Recommended Use	Dominant Mechanisms	Typical Advantages	Evidence Base Compiled Here
Si + Se	Heavy metals (Cd, Pb), salinity, drought, heat; one biotic report. For metal-contaminated or saline soils and Se biofortification.	Represses metal transporters (OsHMA2, OsLCT1, and OsNramp5), induces vacuolar OsHMA3; GSH- and phytochelatin-mediated detoxification; Se accumulation in edible tissues.	Lower grain Cd and Pb; preserved photosynthesis, delayed senescence, higher grain quality and Se.	25 studies (Table 1). Metals 10; salinity 5; drought 4; unstressed 4; heat 1; biotic 1. Interaction never tested; three studies find the combination inferior to single application [137,140,150]; one applies the elements as an inseparable nanocomposite [144].
Si + S	Drought and S-deficient soils; one salinity study, none under heavy metals. For water-limited, S-poor soils.	S supplies cysteine, methionine and glutathione for redox buffering and protein synthesis; Si sustains membrane and chloroplast integrity, osmotic adjustment and nutrient uptake.	Higher water-use efficiency and nutrient utilization; more biomass; better reproductive development and grain or pod yield.	9 studies (Table 2). Unstressed 5; drought 3; salinity 1. Interaction never tested; four full factorial designs [151,162,163,164] would permit it.
Si + Zn	Salinity and Zn-deficient soils; occasional drought and heavy-metal reports. For Zn-poor and saline soils.	Zn acts as an enzyme cofactor and in GA_3_/ABA balance; Si improves K^+^/Na^+^ homeostasis, stomatal integrity and osmotic adjustment.	Higher nutrient- and water-use efficiency, chlorophyll content, productivity and product quality.	10 studies (Table 3). Salinity 3; Zn deficiency 3; unstressed 2; drought 1; metals 1. Interaction never tested; four entries lack a combined treatment [170,174,176,177], and three are confounded with other factors [167,168,171].
Si + Fe	Heavy metals (Cd, Pb, As, and Cr); single salinity and drought reports. For metal-contaminated soils.	Fe-plaque formation on roots; regulation of Fe- and Si-related transporters (OsIRT2, OsLsi1); metal immobilization; maintenance of Fe homeostasis.	Reduced metal uptake and translocation; improved chlorophyll formation, photosynthesis, biomass and antioxidant protection.	8 studies (Table 4). Metals 6; salinity 1; drought 1. Interaction never tested; two entries apply the elements inseparably or in separate experiments [181,186].

Counts refer to the studies compiled in Table 1, Table 2, Table 3 and Table 4 (n = 52) and describe the evidence base of this review, not effect size. No study tested the Si × element interaction statistically, so the mechanisms listed describe processes reported in the cited studies, not demonstrated interaction effects. GA_3_/ABA regulation under Si–Zn rests on a single study.

## Data Availability

No new data were created or analyzed in this study. Data sharing is not applicable to this article.
